# Genomic analysis and biochemical profiling of an unaxenic strain of *Synechococcus* sp. isolated from the Peruvian Amazon Basin region

**DOI:** 10.3389/fgene.2022.973324

**Published:** 2022-11-09

**Authors:** Marianela Cobos, Ruth C. Condori, Miguel A. Grandez, Segundo L. Estela, Marjorie T. Del Aguila, Carlos G. Castro, Hicler N. Rodríguez, Jhon A. Vargas, Alvaro B. Tresierra, Luis A. Barriga, Jorge L. Marapara, Pedro M. Adrianzén, Roger Ruiz, Juan C. Castro

**Affiliations:** ^1^ Laboratorio de Biotecnología y Bioenergética (LBB), Universidad Científica del Perú (UCP), Iquitos, Perú; ^2^ Unidad Especializada de Biotecnología, Centro de Investigación de Recursos Naturales de la UNAP (CIRNA), Universidad Nacional de la Amazonia Peruana (UNAP), Iquitos, Perú; ^3^ Departamento Académico de Ciencias Biomédicas y Biotecnología, Facultad de Ciencias Biológicas, Universidad Nacional de la Amazonia Peruana (UNAP), Ciudad Universitaria de Zungarococha, Iquitos, Perú; ^4^ Instituto de Física de São Carlos, Universidade de São Paulo, São Carlos, Brazil; ^5^ Facultad de Industrias Alimentarias, Universidad Nacional de la Amazonia Peruana (UNAP), Ciudad Universitaria de Zungarococha Iquitos, Iquitos, Perú

**Keywords:** biotechnological exploitation, cyanobacteria, genome analysis, microbial biodiversity, nutraceuticals, biochemical analysis

## Abstract

Cyanobacteria are diverse photosynthetic microorganisms able to produce a myriad of bioactive chemicals. To make possible the rational exploitation of these microorganisms, it is fundamental to know their metabolic capabilities and to have genomic resources. In this context, the main objective of this research was to determine the genome features and the biochemical profile of *Synechococcus* sp. UCP002. The cyanobacterium was isolated from the Peruvian Amazon Basin region and cultured in BG-11 medium. Growth parameters, genome features, and the biochemical profile of the cyanobacterium were determined using standardized methods. *Synechococcus* sp. UCP002 had a specific growth rate of 0.086 ± 0.008 μ and a doubling time of 8.08 ± 0.78 h. The complete genome of *Synechococcus* sp. UCP002 had a size of ∼3.53 Mb with a high coverage (∼200x), and its quality parameters were acceptable (completeness = 99.29%, complete and single-copy genes = 97.5%, and contamination = 0.35%). Additionally, the cyanobacterium had six plasmids ranging from 24 to 200 kbp. The annotated genome revealed ∼3,422 genes, ∼ 3,374 protein-coding genes (with ∼41.31% hypothetical protein-coding genes), two CRISPR Cas systems, and 61 non-coding RNAs. Both the genome and plasmids had the genes for prokaryotic defense systems. Additionally, the genome had genes coding the transcription factors of the metalloregulator ArsR/SmtB family, involved in sensing heavy metal pollution. The biochemical profile showed primary nutrients, essential amino acids, some essential fatty acids, pigments (e.g., all-trans-β-carotene, chlorophyll a, and phycocyanin), and phenolic compounds. In conclusion, *Synechococcus* sp. UCP002 shows biotechnological potential to produce human and animal nutrients and raw materials for biofuels and could be a new source of genes for synthetic biological applications.

## 1 Introduction

Cyanobacteria are an ancient lineage and polyphyletic group of prokaryotes exhibiting oxygenic photosynthesis (Kauff and Büdel, 2011). These microorganisms are the most important primary producers on Earth, inhabiting and playing key roles in a great diversity of aquatic and terrestrial ecosystems exposed to light (Dvořák et al., 2015). Estimates of the current cyanobacterial biodiversity range from 5,301 (AlgaeBase: Listing the World’s Algae, 2022) to 8,000 species (Guiry, 2012).

Due to their extraordinary biodiversity and metabolic diversity, cyanobacteria are potentially valuable for humans in several ways. For example, microalgae could be useful in aquaculture, producing foods, feeds, biofuels, fertilizers, nutraceuticals, secondary metabolites, pigments, and a myriad of bioactive biochemicals (Singh et al., 2005; Udayan et al., 2017; Cobos et al., 2020). In addition, cyanobacteria are isolable and cultivable in laboratory conditions (Ernst, 1991; Badr et al., 2018; Prihantini, 2020; Thilak et al., 2020; Jasser et al., 2022; Singh and Kumar, 2022). Moreover, cyanobacteria have a relatively short doubling time and can produce high biomass volumes (Yu et al., 2015; Jaiswal et al., 2018; Mastropetros et al., 2022; Prabha et al., 2022). These microorganisms also have small genomes readily deciphered at structural and functional levels (Jaiswal et al., 2018; Lin et al., 2019; Kling et al., 2022; Pierpont et al., 2022). Finally, several cyanobacteria are genetically transformable. The genetic transformability of these microorganisms can broaden their spectrum of biotechnological uses (Jaiswal et al., 2018; Jaiswal et al., 2020; Purdy et al., 2022; Santos-Merino et al., 2022; Tan et al., 2022).

Despite all these advantages of cyanobacteria as biotechnological platforms, only a few species have been exploited at commercial levels (Lem and Glick, 1985; Grewe and Pulz, 2012; Yu et al., 2015, 2). Consequently, it is necessary to constantly make bioprospection efforts and basic studies at the biochemical and molecular levels. These approaches will help us discover cyanobacterial strains with desirable phenotypic and genotypic traits. These desirable traits include the fastest growth, highest productivity, metabolic diversity, and genetic transformability (Thajuddin and Subramanian, 2005; Al-Haj et al., 2016; Selão, 2022). In this context, the present study shows the genome features and the biochemical profile of *Synechococcus* sp. UCP002, a cyanobacterium isolated from the Peruvian Amazon Basin region.

## 2 Materials and methods

### 2.1 Sample collection

Water samples were collected horizontally along the water surface of the Amazon River using a 20-μm plankton net (Continental TEM, Lima, Peru) using a boat towing method. The plankton net was held horizontally at 20-cm depth and dragged ∼100 m from the geographic coordinates 03°41′0.6″ S, 73°14′8.9″ W to 03°40′57″ S, 73°14′08″ W. Sterile, screw-cap, wide-mouth 500-ml glass bottles were used to collect and transport the water samples at ∼8°C in dark conditions.

### 2.2 Isolation, culture, growth profile, and harvest of the cyanobacterium

A total of 50 milliliters of the filtered water sample was homogenized with 50 ml of BG-11 medium (Allen, 1968). The cyanobacterial cells were cultured for 4 weeks in a controlled culture room at 25.27 ± 0.06°C with 12:12-h light–dark cycles using 265 ± 10 μE m^−2^ s^−1^ intensity of a 50-W LED-based white light source (Wellmax, Samsung) with continuous bubbling of air and shaking the cultures at 180 rpm. After the initial cultivation of the mixed cultures, unicellular cyanobacteria were subjected to isolation by the cell washing method (Richmond and Hu, 2013) and by repeat sub-culturing and plating on a solid BG-11 agar medium.

After isolation, cyanobacterial cells were inoculated and cultured in 50 ml of the BG-11 medium. According to the growth of the cyanobacteria, the culture volume was increased gradually to obtain 1 L of culture.

To determine the cyanobacterium growth profile, an aliquot of the culture was taken and subcultured in triplicate at a final volume of 200 ml in Erlenmeyer flasks (250 ml) in a controlled culture room at 38.21 ± 0.76°C in 12:12-h light–dark cycles using 500 μE m^−2^ s^−1^ intensity of a 50-W LED-based white light source (Wellmax, Samsung), with continuous bubbling of air and shaking the cultures at 180 rpm. The initial absorbance at 730 nm (A_730_ = 0.104 ± 0.05) was determined by spectrophotometric analysis using a NanoDrop 2000 spectrophotometer (Thermo Fisher Scientific, United States). The growth of the cyanobacterium was monitored every day for 12 days by recording its absorbance at 730 nm and the dry weight of the cyanobacterial biomass. To determine both measures, aliquots of 2.1 ml were obtained from the culture every day at the sixth hour after the start of the illumination phase of the photoperiod. To determine the dry weight of the cyanobacterial biomass, 2 ml of the culture was harvested by centrifugation at 10,000 × *g* for 5 min at 4°C, and the pellets were washed two times with 1 ml of a physiological saline solution. Finally, the samples were dried in an oven at 70°C for 24 h, and the dry weight was measured gravimetrically using an analytical balance Kern ABJ 220-4NM (Kern and Sohn GmbH, Balingen, Germany).

To determine the cyanobacterium-specific growth rate (µ) and the doubling time (*t*_d_, generation time in hours), an aliquot of the culture was subcultured in triplicate at a final volume of 200 ml in Erlenmeyer flasks (250 ml) in a controlled culture room at 38.21 ± 0.76 °C in constant illumination using 500 μE m^−2^ s^−1^ intensity of a 50-W LED-based white light source (Wellmax, Samsung), with continuous bubbling of air and shaking the cultures at 180 rpm. The initial absorbance at 730 nm was 0.085 ± 0.007. The growth of the cyanobacterium was monitored recording absorbances at 730 nm every 2 hours for 14 h. Based on the absorbance data on this period of time, the *μ* and *t*_d_ growth parameters were computed using the following equations:

$$Specific\;growth\;rate\;\left(\mu \right)=\frac{\mathrm{Ln}\left(A_{f}\right)-\mathrm{Ln}\left(A_{i}\right)\;}{t_{f}-t_{i}},$$

$$Doubling\;time\;\left(t_{d}\right)=\frac{\mathrm{Ln}\left(2\right)}{\mu },$$

where *A* is the absorbance at 730 nm (A_730_) at the final (_*f*_) or initial (_*i*_) time (*t*).

For the biochemical analysis, the cyanobacterial cells were harvested during the exponential growth phase of cultures at the sixth hour after the start of the illumination period. The culture was transferred to 50-ml conical-bottom centrifuge tubes and centrifuged at 2,000×*g* for 15 min at 4°C to harvest the cyanobacterial cells. The obtained cyanobacterial biomass was rinsed three times with 40 ml of sterilized ultrapure water, centrifuged again in the aforementioned conditions, and the supernatants were discarded.

### 2.3 Morphological and molecular identification of the isolated cyanobacterium

The isolated cyanobacterium was preliminarily identified using standard microscopic morphological characteristics. Also, the autofluorescence emitted was recorded using a Carl Zeiss fluorescence microscope. Microphotographs were obtained using a digital camera AxioLab.A1 AxioCam ERc real-time 5 s. Images were obtained at a magnification of ×400 with visible light and epifluorescence (excitation 510–560, emission 590). The average cell size (length and width) of the isolated cyanobacterium was estimated from 100 cells by ZEN 2012 × 32 blue software (Carl Zeiss, Jena, Germany).

For molecular identification, a phylogenomic analysis was conducted with 31 conserved proteins (Wu and Eisen, 2008). These conserved proteins were retrieved from complete genomes of *Synechococcus* sp. UCP002 and 43 cyanobacteria species. Furthermore, these proteins were concatenated, aligned, and trimmed using the tools of Geneious Prime^®^ 2022.2.2 (Kearse et al., 2012). Finally, a maximum likelihood tree with 100 bootstrap replicates was inferred using MEGA 11 (Tamura et al., 2021). The Le–Gascuel model (Le and Gascuel, 2008) of amino acid substitution was selected based on the likelihood test. A discrete gamma distribution was used to model evolutionary rate differences among sites (four categories (+G, parameter = 0.7090)) and a proportion of invariable sites.

### 2.4 Genomic DNA purification, library preparation, and shotgun sequencing

Genomic DNA was extracted from 200 mg of cyanobacterial biomass using a modified CTAB method (Cobos et al., 2017) and purified using the DNeasy^®^ PowerSoil Pro Kit (QIAGEN, Germany), following the manufacturer’s instructions. The quality and quantity of the purified genomic DNA were determined by spectrophotometric analysis using a NanoDrop 2000 spectrophotometer (Thermo Fisher Scientific, United States). DNA integrity and purity were evaluated by electrophoretic analysis on agarose gels (Sambrook and Russell, 2006). DNA quantity was determined with the Qubit™ dsDNA BR Assay Kit using a Qubit™ 4 Fluorometer (Thermo Fisher Scientific, United States).

Libraries were prepared using the Nextera XT DNA Library Preparation Kit (Illumina, United States), following the manufacturer’s instructions. Purified DNA was fragmented and tagged using a tagmentation process. Index adapters were ligated to the tagmented DNA using a limited-cycle PCR program. The libraries were cleaned up by 0.8x Agencourt^®^ AMPure XP bead purification (Beckman Coulter, United States). The sizes of the libraries were determined using an Agilent High Sensitivity DNA Kit by Agilent 2100 Bioanalyzer microfluidic electrophoresis (Agilent Technologies, United States). Finally, the libraries were quantified using the Qubit™ dsDNA HS Assay Kit (Thermo Fisher Scientific) and paired-end sequenced with the Illumina NexSeq 550 platform.

### 2.5 Bioinformatic analysis

Raw Illumina paired-end reads were uploaded as FASTQ files and analyzed using the Galaxy (Jalili et al., 2020) and KBase (Arkin et al., 2018) platforms. From raw sequences, the high-quality reads were obtained with Trimmomatic v0.38.1 (Bolger et al., 2014), and read qualities were evaluated with FastQC v0.11.9 (Andrews, 2010).

To verify that the cyanobacterial strain is axenic or unaxenic, the clean reads were taxonomically assigned with GOTTCHA2 software v2.1.7 (Freitas et al., 2015). Because the results generated by GOTTCHA2 software show associated bacteria, the *de novo* assembly of the complete genome of *Synechococcus* sp. UCP002 was conducted, following the bioinformatic approaches described as follows.

Short sequences were subjected to the first round of the *de novo* assembly using the assemblers IDBA-UD v1.1.3 (Peng et al., 2012), MEGAHIT v1.2.9 (Li et al., 2015), metaSPAdes v3.15.3 (Nurk et al., 2017), SPAdes v3.15.3 (Bankevich et al., 2012), and Velvet v1.2.10 (Zerbino, 2010). Qualities and assembly parameters were assessed with QUAST v4.4 (Gurevich et al., 2013). Next, the second round of the *de novo* assembly was conducted using the totality of contigs obtained with the five assemblers. For this process, the contigs were elongated and assembled (scaffolding) using the mapper and the *de novo* assembler tools of Geneious Prime^®^ 2022.2.2 (Kearse et al., 2012). Furthermore, the third round of the assembly was conducted using the MaSuRCA genome assembler v3.2.9 (Zimin et al., 2013) using a combination of the generated contigs and scaffolds and the high-quality short reads. In addition, to reconstruct the draft genome, contigs and scaffolds were binned using CONCOCT v1.1 (Alneberg et al., 2014), MaxBin2 v2.2.4 (Wu et al., 2016), and MetaBAT2 v1.7 (Kang et al., 2019). Binned contigs and scaffolds were optimized by dereplication, aggregation, and scoring approaches using the DAS Tool v1.1.2 (Sieber et al., 2018). Taxonomic assignments of the optimized bins were based on the Genome Taxonomy Database (GTDB; https://gtdb.ecogenomic.org) (Parks et al., 2022) using GTDB-Tk v1.7.0 (Chaumeil et al., 2020). The bin containing contigs and scaffolds derived from cyanobacteria (*Synechococcus* sp.) was extracted as an assembly using the BinnedContigs tool v1.0.2. Furthermore, the complete genome was obtained by re-assembling the contigs and scaffolds and the clean paired-end reads by Unicycler v0.4.8.0 software (Wick et al., 2017). Finally, prior to downstream annotation analysis, coverage, quality, contamination, and completeness of the genome were evaluated using the Geneious mapper (Kearse et al., 2012), CheckM v1.0.18 (Parks et al., 2015), and BUSCO v5.3.2 (Simão et al., 2015), respectively. Additionally, the prediction of plasmid sequences in contigs and scaffolds was conducted by PlasFlow v1.0 software (Krawczyk et al., 2018), and its assembly was completed by NOVOPlasty v4.3.1 software (Dierckxsens et al., 2017).

The circular genome map of *Synechococcus* sp. UCP002 was aligned with its closest genetic neighbors using the Proksee server (https://proksee.ca/). Also, the circular maps of the plasmids were generated using the same online server.

The assembled genome was functionally annotated using the following tools: Bakta v1.5.0 (Schwengers et al., 2021), dFast v1.6.0 (https://dfast.ddbj.nig.ac.jp/) (Tanizawa et al., 2018), DRAM v0.1.0 (Shaffer et al., 2020), KAAS (https://www.genome.jp/kegg/kaas/) (Moriya et al., 2007), Prokka v1.14.5 (Seemann, 2014), and RASTtk v1.073 (Brettin et al., 2015). Additionally, the CRISPR–Cas elements in the genome and plasmids were identified by the online software application CRISPRCasFinder (Couvin et al., 2018).

The genes coding the enzymes of the phenylpropanoid/flavonoid biosynthetic pathway were found by conducting a local BLAST search (Camacho et al., 2009) according to Del Mondo et al. (2022). Sequences of 29 core enzymes acting in the phenylpropanoid/flavonoid biosynthetic pathway of plants and cyanobacteria from KEGG were used as queries to detect ortholog sequences in the complete genome of *Synechococcus* sp. UCP002. The sequences with the best hit matches for each core enzyme were retained. Furthermore, the protein sequences were used to make a BLAST search against the UniProt TrEMBL protein database (UniProt Consortium, 2021).

The protein sequences coded by the genes *smt*B of *Synechococcus* sp. UCP002 and some cyanobacterial species (*Synechococcus* sp. PCC 6312 (WP_015123347), *Calothrix* sp. PCC 7507 (WP_01512714), *Leptolyngbya* sp. PCC 6406 (WP_008314625), *Oscillatoria nigro-viridis* (WP_01574920), *Nostoc* sp. PCC 7107 (WP_015114276), and *Anabaena* sp. PCC 7108 (WP_016952607)) were aligned using the Alignment tool of Geneious Prime^®^ 2022.2.2.

The prediction of the three-dimensional structure of the proteins SMTB1, SMTB2, and SMTA involved in the metal-responsive cyanobacterial expression system of *Synechococcus* sp. UCP002 was realized using the SWISS-MODEL server (https://swissmodel.expasy.org/). The three-dimensional models for the two proteins of the metal-sensing transcriptional repressors (SMTB) were based on the cyanobacterial metallothionein repressor from *Synechococcus elongatus* PCC 7942 (PDB accession: 1SMT). The model corresponding to metallothionein was based on the cyanobacterial metallothionein SMTA from *Synechococcus elongatus* PCC 7942 (PDB accession: 1JJD).

### 2.6 Biochemical analysis of the cyanobacterial biomass

For proximate composition analysis, the cyanobacterial biomass was dried in an oven at 70°C. The dried biomass was measured gravimetrically using an analytical balance Kern ABJ 220-4NM (Kern and Sohn GmbH, Balingen, Germany). Total lipids were extracted following the Bligh and Dyer method (Bligh and Dyer, 1959) and quantified gravimetrically using a semi-micro analytical balance (Sartorius, MSU225S-000-DU, Foster City, CA, United States). Total carbohydrates were determined using a colorimetric method (DuBois et al., 1956). The protein content was measured following the Hartree approach (Hartree, 1972). The ash content was determined by thermogravimetry (AOAC, 1990) using a Thermolyne™ F6010 muffle furnace (Thermo Fisher Scientific, Waltham, MA, United States) set at 550°C for 16 h.

For pigment analysis (all-trans-β-carotene, lutein, and chlorophyll a), 40 mg of the freeze–dried cyanobacterial biomass was homogenized with 5 ml of acetone 100%. The acetonic extract was filtered onto 0.45-μm PTFE membrane filters to remove cells and cell debris. Next, 20 μl of pigment solutions were resolved using a Hitachi Elite LaChrom HPLC System (Hitachi High Technologies, San Jose, CA, United States) equipped with an L-2200 autosampler, L-2130 HTA pump, L-2350 column oven, L-2455 diode array detector, L-2485 fluorescence detector, and a 150 × 4.6 mm x 5 μm MilliporeSigma™ LiChroCART™ LiChrosorb™ RP-8 C8 Reversed Phase HPLC Column (Merck, Darmstadt, Germany). The HPLC system was programmed to run under the following conditions: column temperature: 25°C, flow rate: 1 ml/min, and absorbance monitoring at 450 nm. A ternary mobile phase consisted of (A) 100% methanol, (B) methanol: ammonium acetate 0.5 N (80:20), and tetrahydrofuran. The following gradient elution was employed: 0 min: (0% A, 100% B, and 0% C), 5 min (98% A, 0% B, and 2% C), 42.2 min (80% A, 0% B, and 20% C), 26 min (98% A, 0% B, and 2% C), 34 min (0% A, 100% B, and 0% C), and 30 min of column equilibration (100% A, 0% B, and 0 %C). EZChrom Elite software v3.2.1 (Agilent Technologies, Santa Clara, CA, United States) was used for data acquisition and analysis, compared with the chromatographic profiles of authentic standards of all-trans-β-carotene, lutein, and chlorophyll a (Sigma-Aldrich, Saint Louis, MO, United States).

For the determination of the total content of phycocyanin *in vivo* (c-phycocyanin [CPC] + allophycocyanin [APC]), the absorbances at 620 and 652 nm of an aliquot of the cyanobacterial culture in the logarithmical growth phase were recorded using a NanoDrop 2000 spectrophotometer (Thermo Fisher Scientific, United States). Finally, the CPC and APC contents were determined with the following equations (Bennett and Bogorad, 1973; Chaiklahan et al., 2012):

$$CPC\;\left(mg.\;{mL}^{-1}\right)=\frac{A_{620}-\left(0.474\;x\;A_{652}\right)}{5.34},$$

$$APC\;\left(mg.\;{mL}^{-1}\right)=\frac{A_{652}-\left(0.208\;x\;A_{620}\right)}{5.09}.$$

For total phenolic content (TPC) analysis, first, a hydromethanolic extract was obtained from 100 mg of the cyanobacterial dry biomass using an approach previously described (Cobos et al., 2020). Furthermore, the total phenolic content was estimated by the Folin–Ciocalteu method (Velioglu et al., 1998) based on a standard curve from 10 to 100 μM of gallic acid (3,4,5-trihydroxy benzoic acid) (Sigma-Aldrich, Germany). Results of the total phenolic content were expressed as gallic acid equivalents (mg GAE. g^−1^ of cyanobacterial biomass dry weight [cbdw]).

For fatty acid analysis, first, fatty acid methyl esters (FAMEs) were obtained following an acid-catalyzed methanolysis/methylation approach (Ichihara and Fukubayashi, 2010); furthermore, FAMEs were resolved by using a gas chromatographic method (Cobos et al., 2020). FAMEs were identified by comparing the retention time of the peaks with a known standard mixture (Nu-Chek Prep, Elysian, MN, United States). Also, each sample was mixed with tricosanoic acid methyl ester (Sigma-Aldrich, Saint Louis, MO, United States) as the internal standard. Finally, generated chromatograms were analyzed with Galaxie™ Chromatography Data System software v1.9.3.2 (Agilent Technologies, Santa Clara, CA, United States).

For amino acid analysis, total proteins were subjected to acid hydrolysis (Hirs et al., 1954); furthermore, amino acids obtained by hydrolysis and amino acid standards (Sigma-Aldrich, Saint Louis, MO, United States) were derivatized with 6-aminoquinolyl-N-hydroxysuccinimidyl carbamate, following instructions of the AccQ-Fluor Reagent Kit (Waters Corporation, Milford, MA, United States). Derivatized amino acids were identified and quantified using an HPLC method (Cohen and Michaud, 1993). All the described biochemical analyses were carried out in triplicate, and data are expressed as the mean ± SD.

## 3 Results and discussion

### 3.1 Isolation, growth profile, and identification

**FIGURE 1:**
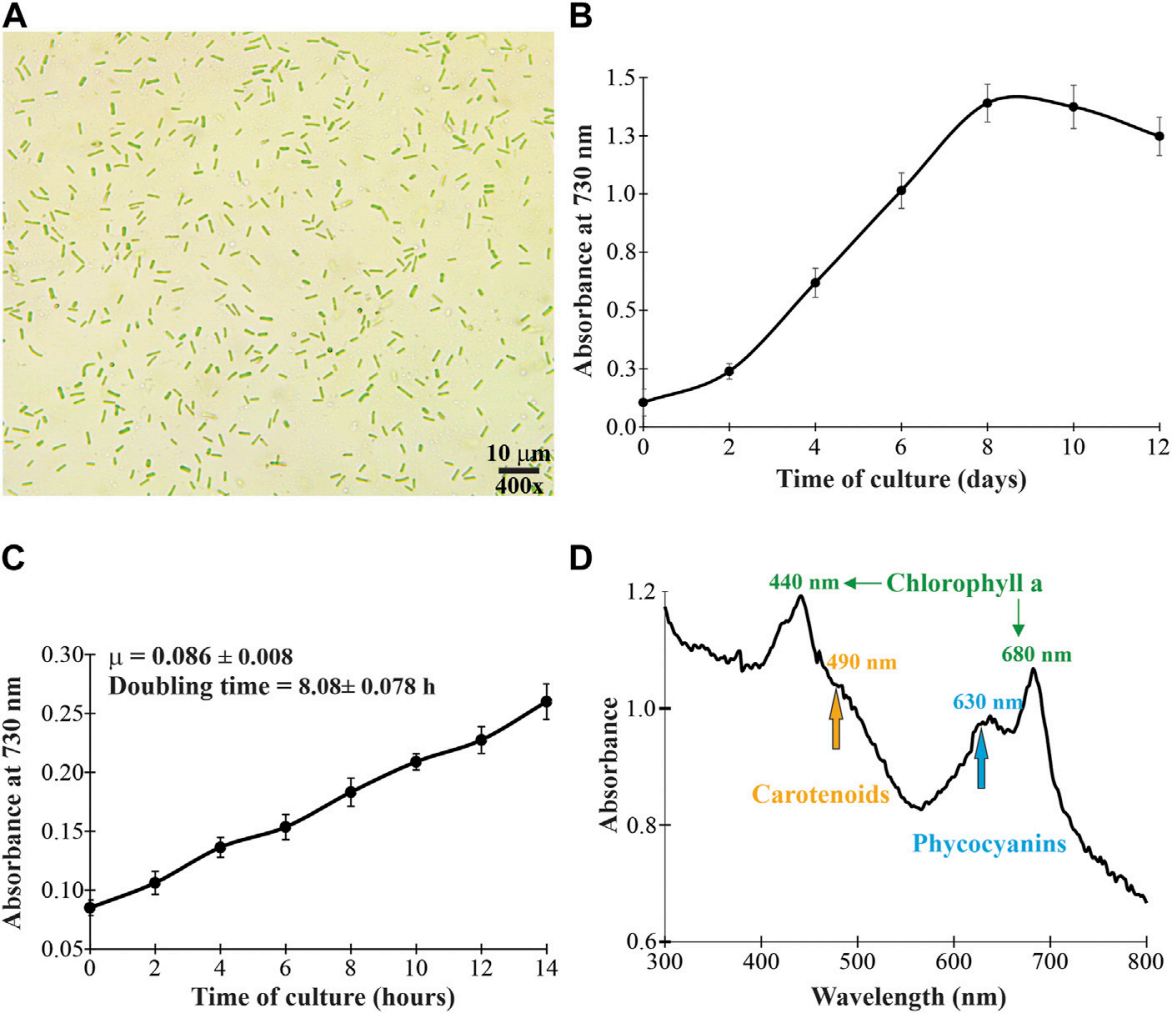
Microscopic morphology **(A)**, growth profile **(B)**, specific growth rate and doubling time **(C)**, and *in vivo* absorption spectrum **(D)** of the cyanobacterium *Synechococcus* sp. UCP002 isolated from the Peruvian Amazon Basin region.

In this work, we report the discovery of the cyanobacterial strain *Synechococcus* sp. UCP002 from the Peruvian Amazon Basin. The unicellular cyanobacterium strain showed a typical growth profile with the lag, logarithmic, and stationary phases in an interval time of 12 days (Figure 1, Supplementary Figure S1). The average values for the specific growth rate and the cell doubling time in constant illumination for 14 h were estimated at 0.086 ± 0.008 *μ* and 8.08 ± 0.78 h, respectively (Figure 1). It is difficult to compare this cell doubling time value with reports for other cyanobacterial strains due to differences in culture conditions (e.g., photoperiod, light intensity, temperature, and use or number of photobioreactors). Some doubling time values are 4.9 h for *Synechococcus elongatus* PCC 7942 (Ungerer et al., 2018a) and 11.8 h for *Synechococcus* sp. AMC149 (Mori et al., 1996; Kondo et al., 1997). Also, some studies report the existence of fast-growing strains of *S. elongatus.* For example, the strains PCC 11801 and PCC 11802 isolated from Powai Lake (India) have doubling times of 2.3 h (Jaiswal et al., 2018) and 2.8 h (Jaiswal et al., 2020, 11801), respectively. Similarly, *Synechococcus* sp. PCC 11901 isolated from the Johor Strait (Singapore) has a doubling time of ≈2.0 h (Włodarczyk et al., 2020). Finally, *S. elongatus* UTEX 2973 is up-to-date the fastest-growing cyanobacterium with a doubling time of 1.5 h (Yu et al., 2015; Ungerer et al., 2018b).

To corroborate the cyanobacterial nature of the strain, first, the whole-cell absorbance spectrum was recorded in the UV-visible range (from 280 to 800 nm). The whole-cell absorbance showed a peak at 635 nm, which corresponds to C-phycocyanin (Abalde et al., 1998; Gupta and Sainis, 2010; Sonani et al., 2017). Additionally, the whole-cell absorbance showed peaks at 440 and 680 nm. This absorbance pattern is in agreement with the typical absorbance of chlorophyll a. Also, the whole-cell absorbance showed a less defined peak at 483 nm. This peak corresponds to carotenoids (Figure 1). Microscopically, the cyanobacterium is unicellular with rod-shaped morphology (Figure 1). The cyanobacterium had an average length of 3.61 ± 0.54 μm (from 2.49 to 4.34 μm) and a width of 1.42 ± 0.31 μm (from 1.06 to 2.10 μm). These cellular dimensions are similar to those of *Synechococcus* sp. 6,312 (2.7 × 1.3 μm) (Allen and Stanier, 1968) and some strains of *Synechococcus elongatus* (Jaiswal et al., 2018; Jaiswal et al., 2020). Together, these typical morphological characteristics corroborate that the cyanobacterium belongs to the Synechococcaceae family.

**FIGURE 2:**
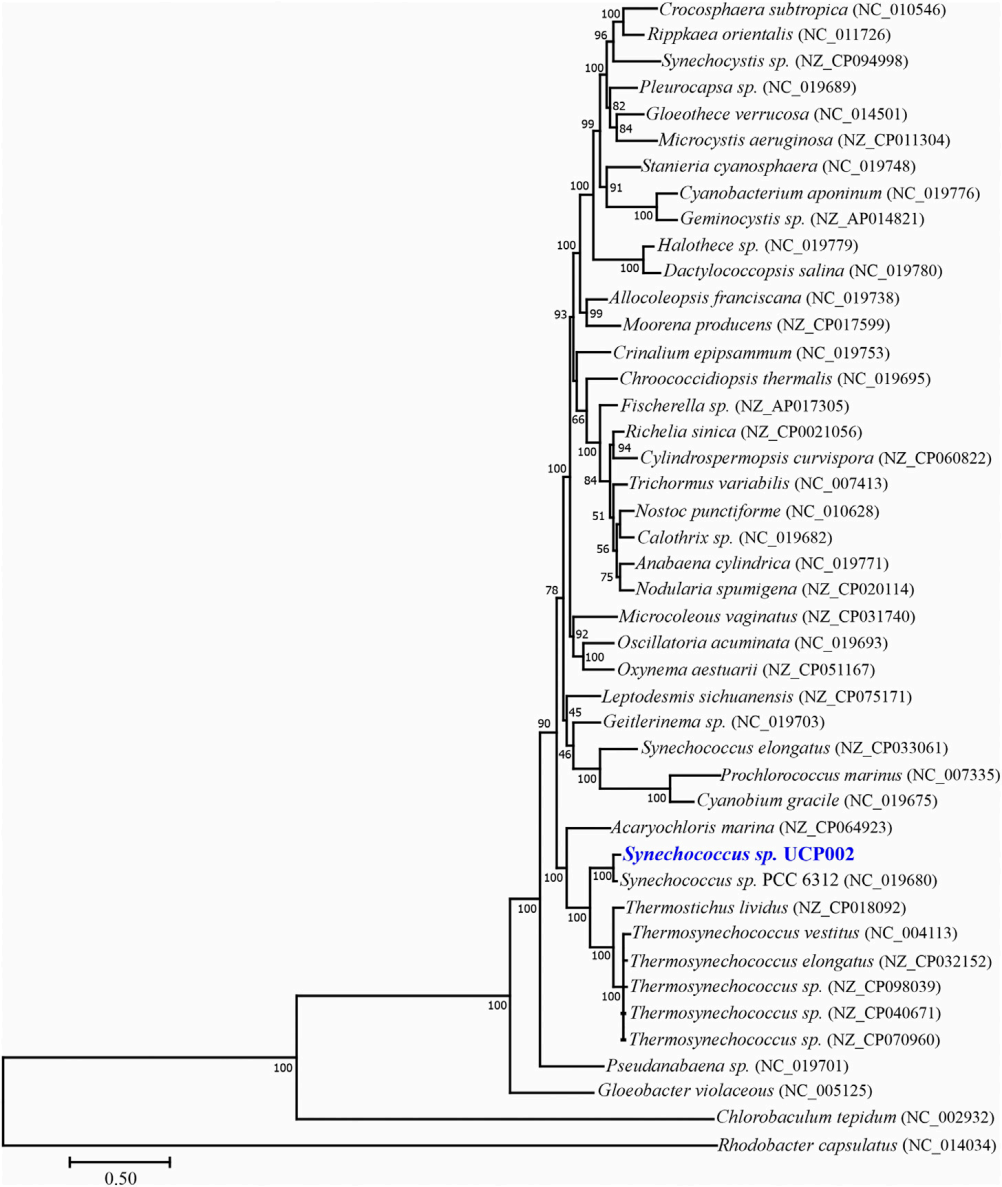
Maximum likelihood phylogenomic tree with bootstrap support inferred based on 31 conserved proteins.

Finally, based on the phylogenomic analysis of 31 conserved proteins (Wu and Eisen, 2008), the isolated strain forms a clade with cyanobacteria of different genera. These cyanobacterial genera include *Acaryochloris, Synechococcus*, *Thermostichus*, and *Termosynechococcus* (Figure 2). But *Synechococcus* sp. UCP002 showed the highest genetic similitude with *Synechococcus* sp. PCC 6312. *Synechococcus* sp. PCC 6312 is a fresh-water cyanobacterium isolated from California (United States) in 1963 (https://www.ncbi.nlm.nih.gov/biosample/SAMN02261337), and its complete genome was sequenced by the CyanoGEBA Sequencing Project (https://www.ncbi.nlm.nih.gov/bioproject/158717). *Synechococcus* sp. PCC 6312 shows similitude in shape (rod-shaped) and size (Allen and Stanier, 1968) with *Synechococcus* sp. UCP002. In addition, this cyanobacterium strain can intracellularly biomineralize amorphous calcium carbonate. These calcium carbonate inclusions are located mostly in the cellular poles of the cyanobacterium (Benzerara et al., 2014; De Wever et al., 2019).

### 3.2 Genome analysis

#### 3.3.1 Genome assembly

**FIGURE 3:**
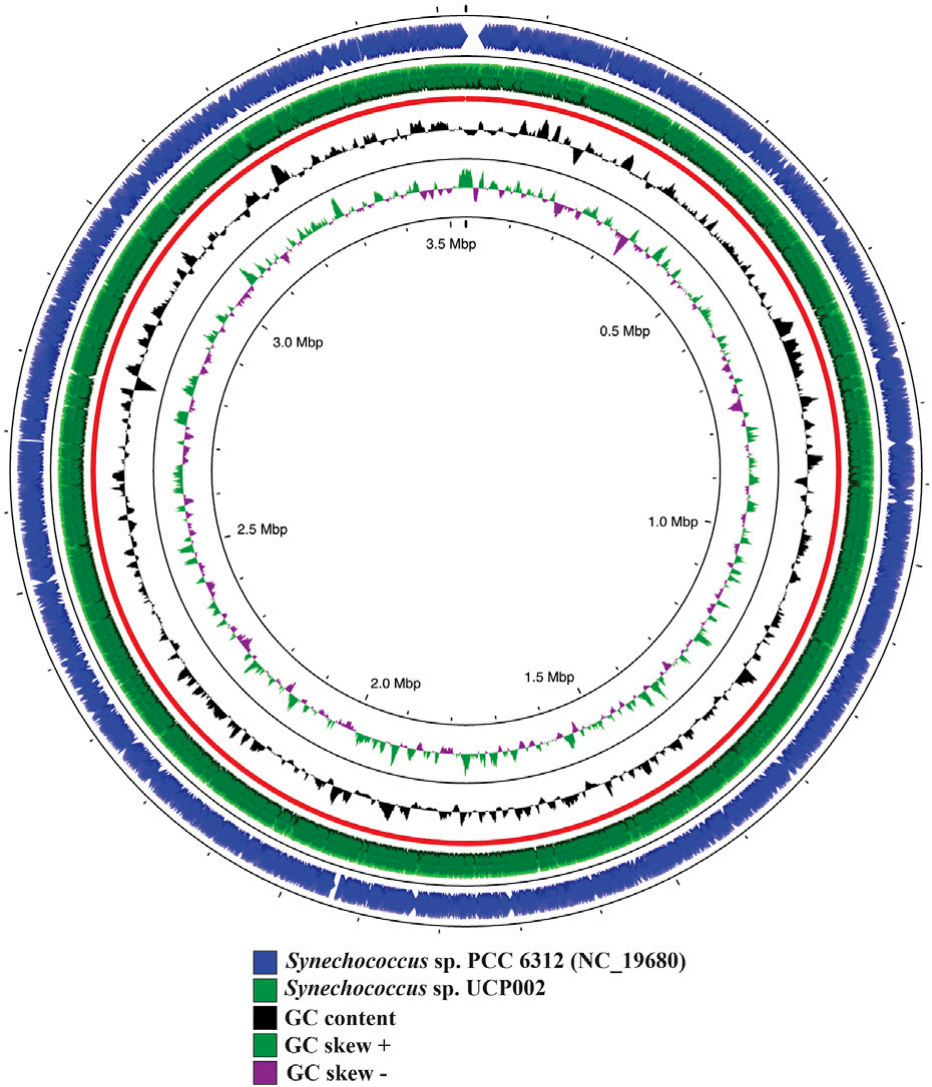
Genome map of *Synechococcus* sp. UCP002 compared with the closest relative (*Synechococcus* sp. PCC 6312). The circular map also shows the GC content and GC skew (+/−). The gap regions show no overlapping regions with the closest neighbor.

A total of 4,838,556 reads were *de novo* assembled to generate the complete genome of *Synechococcus* sp. UCP002. The complete genome had a size of ∼3.53 Mb and a GC content of 47.87% (Figure 3). The GC content and the genome size recorded fit in the range of values reported for cyanobacteria with complete genomes of the genus *Synechococcu*s (Supplementary Table S1) (Sugita et al., 2007; Jaiswal et al., 2018; Jaiswal et al., 2020, 11801; Kling et al., 2022; Pierpont et al., 2022, 1). Also, we assembled and annotated six plasmids with sizes ranging from 24.44 to 200.03 kbp (Supplementary Figure S2).

According to the analysis, the complete genome had a high coverage and was characterized by its high quality. The coverage was ∼200x, and the BUSCO results indicate that more than 97% of the 40 core genes were complete and single-copy genes, and only a very low fraction was missing. In addition, the CheckM results showed that the genome had completeness of more than 99% and a low contamination level (0.35%).

#### 3.3.2 Functional annotation of the genome

The complete genome of *Synechococcus* sp. UCP002 contained 3,324–3,640 predicted genes (Table 1). Of the total genes, from 3,280 to 3,596 were protein-coding genes with an average protein length of 305 amino acid residues. Of these protein-coding genes, those from 1,920 to 2,407 were associated with known functions. Of the protein-coding genes with known functions, those from 756 to 948 were enzyme-coding genes. Also, from the total protein-coding genes, those from 903 to 1,752 were hypothetical protein-coding genes. In addition, in the draft genome were recognized seven ncRNA, nine ncRNA regions, two genes coding rRNA (16 and 23S), 41 genes coding tRNAs, one gene coding tmRNA, and two CRISPR–Cas systems. Also, CRISPR–Cas systems were identified in four (pSUCP002.1, pSUCP002.2, pSUCP002.3, and pSUCP002.6) of the six plasmids of *Synechococcus* sp. UCP002 (Supplementary Table S2). CRISPR loci and Cas (CRISPR-associated) operons together (CRISPR–Cas system) constitute a heritable molecular adaptive immune system found in many bacterial and archaea species (Barrangou et al., 2007; Sorek et al., 2008). In cyanobacteria, the CRISPR–Cas systems are classified into class 1 (with types I and III) and class 2 with type V (Pattharaprachayakul et al., 2020).

**TABLE 1:** Results of the functional annotation of the complete genome of *Synechococcus* sp. UCP002 using Bakta, dFast, Prokka, and RASTtk tools.

	Bakta	dFast	Prokka	RASTtk
Total gene count	3,371	3,324	3,354	3,640
CRISPR array	2	2	2	2
Protein-coding genes	3,310	3,280	3,310	3,596
Number of genes with EC number	948	943	756	942
RNA	61	44	44	44
ncRNA	7	—	—	—
ncRNA regions	9	—	—	—
rRNA	3	2	2	2
tRNA	41	41	41	41
tmRNA	1	1	1	1
Proteins associated with known function	2,407	1,920	1,558	2,111
Hypothetical proteins	903	1,360	1,752	1,566
Coding ratio (%)	85.9	84.9	85.6	86.7

This CRISPR–Cas system protects the prokaryotic cells from invading bacteriophages and conjugative plasmids (Sorek et al., 2008; Marraffini and Sontheimer, 2010). Recent investigations have shown that the CRISPR–Cas system has a widespread distribution in the phylum Cyanobacteria. However, marine cyanobacteria of the *Synechococcus* and *Prochlorococcus* subclade do not have this interference system (Cai et al., 2013). This result is apparently paradoxical because these marine cyanobacteria live in an environment with abundant and diverse cyanophages (Suttle and Chan, 1994; Lu et al., 2001).

Actually, the CRISPR–Cas system has emerged as an effective and versatile tool for genetic modification in cyanobacteria of the genus *Synechococcus*. Wendt et al., using targeted genome editing and enrichment outgrowth, created a new strain of *Synechococcus* elongatus 2973-T that was both naturally transformable and fast-growing (Wendt et al., 2022). Also, some researchers demonstrated that the CRISPR–Cas tool can be used for the metabolic engineering of cyanobacteria of the genus *Synechococcus* (Gordon et al., 2016; Racharaks et al., 2021). Consequently, this modern genetic tool could be used to generate genetically improved strains of *Synechococcus* sp. UCP002.

In addition to the CRISPR–Cas system, the genome of *Synechococcus* sp. UCP002 and most of its plasmids contain other prokaryotic defense systems (Supplementary Table S2). These include the restriction and modification system (RM system), the toxin–antitoxin system (TA system), and the DNA phosphorothioate system (PT). The RM system includes genes coding restriction enzymes (types I, III, and IV) and DNA methyltransferases (e.g., *dam*, *yhdj*, and *dcm*). The TA system has genes coding several key proteins of the type II TA system (e.g., *maz*F, *pri*F, *yef*M, and *hig*A-1) and their related factors (i.e., *fts*Z, *mre*B, and *glt*X). Finally, the PT system has genes coding the sulfur modification proteins (e.g., *isc*S, *dnd*B, *dnd*C, and *dnd*D) and genes coding the DNA phosphorothioation-dependent restriction proteins such as *dpt*F, *dtp*G, and *dtp*H. The PT system modifies the DNA backbone and constitutes a protective epigenetic system with multiple functions (i.e., antioxidant, restriction-modification, and virus resistance properties). The occurrence of *dnd* genes and gene clusters is common in the genomes of archaea and bacteria, including cyanobacteria of the orders Nostocales and Synechococcales (Jian et al., 2021). Together, these prokaryotic defense systems are an evolutive response system of bacteria and archaea against the great diversity of genetic parasites. Consequently, in these microorganisms, an important fraction of the genetic information participates in antiparasitic defense. These antiparasitic defense systems use several strategies, including innate immunity (RM system), adaptive immunity (CRISPR–Cas system), dormancy induction, or programmed cell death (TA system) (Makarova et al., 2013; Koonin et al., 2017).

**FIGURE 4:**
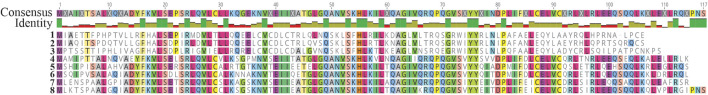
Alignment of protein sequences coded by the gene *smt*B of the representative cyanobacterial species. 1: SmtB1 of *Synechococcus* sp. UCP002, 2: smtB1 of *Synechococcus* sp. PCC 6312 (WP_015123347), 3: smtB of *Calothrix* sp. PCC 7507 (WP_01512714), 4: smtB2 of *Synechococcus* sp. UCP002, 5: smtB of *Leptolyngbya* sp. PCC 6406 (WP_008314625), 6: smtB of *Oscillatoria nigro-viridis* (WP_01574920), 7: smtB of *Nostoc* sp. PCC 7107 (WP_015114276), and 8: smtB of *Anabaena* sp. PCC 7108 (WP_016952607). The alignment was conducted using the Alignment tool of Geneious Prime^®^ 2022.2.2.

Two genes potentially useful for the development of biosensors for heavy metal pollution were identified in the genome of *Synechococcus* sp. UCP002, which are denominated *smt*B1 and *smt*B2. These genes code transcription factors of the metalloregulator ArsR/SmtB family. These transcription factors are negative regulators for the expression of the gene *smt*A, also identified in the genome, which codes cysteine-rich metallothionein that is able to sequester metal ions, such as cadmium, copper, and zinc (Cavet et al., 2003). To corroborate the identity of the proteins encoded by *smt*B1 and *smt*B2, we conducted additional *in silico* analysis by predicting their tridimensional structure (Supplementary Figure S3) and by aligning their amino acid sequences (Figure 4). According to this analysis, the two proteins fit with known proteins involved in the functions described. Together, these genes constitute a metal-responsive expression system (Turner et al., 1996; Busenlehner et al., 2003; Ma et al., 2009). This system is commonly used to develop biosensors to detect environmental pollution with heavy metals (Hui et al., 2021), and consequently, it will be necessary to conduct additional studies to verify the reliability of the genes *smt*B1 and *smt*B2 of *Synechococcus* sp. UCP002 in engineering biosensors for the detection of heavy metal pollution in our region.

### 3.3 Biochemical profiling

According to the bioinformatic analysis, 54% and 46% of the sequencing reads correspond to *Synechococcus* sp. PCC 6312 and the associated bacteria (e.g., Proteobacteria and Dietzia), respectively (Supplementary Figure S4). Consequently, the biochemical profile represents the values of the cyanobacterium and its associated bacteria.

#### 3.1.1 Proximate composition, pigments, and total phenolic content

**FIGURE 5:**
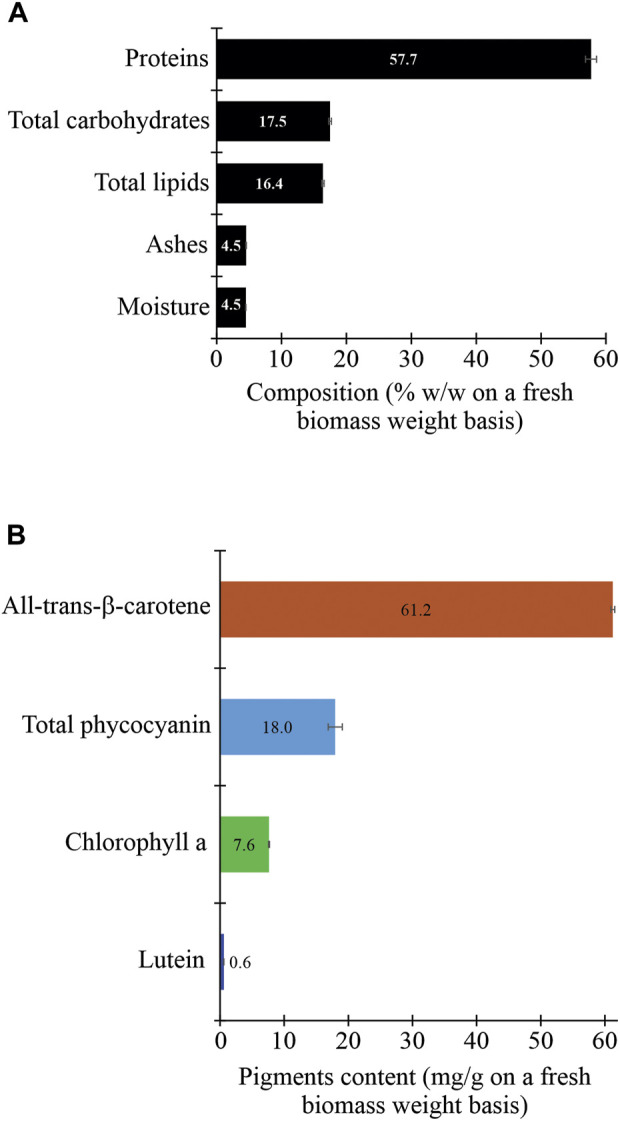
Proximate composition (proteins, carbohydrates, lipids, ashes, and moisture) **(A)** and pigment content **(B)** of the fresh biomass of the cyanobacterium *Synechococcus* sp. UCP002 isolated from the Peruvian Amazon Basin region.

The proximate composition of the unaxenic cyanobacterium showed significant differences in its organic and inorganic contents. Between the organic biomolecules, the proteins were the most abundant (>57%). The second most abundant biomolecules were carbohydrates (>17%), followed by lipids (∼16%). In contrast, the inorganic content, constituted by ashes and moisture, was very low (<5%) (Figure 5). A similar composition of these biomolecules was previously reported for some cyanobacterial strains of the *Arthrospira* genera and microalgae isolated from the Peruvian Amazon Basin region (Cobos et al., 2020). It is necessary to consider that the biochemical composition of the cyanobacterial biomass depends on biotic and abiotic factors. The biotic factors include the growth phase, the associate microorganisms (e.g., yeasts, microalgae, and bacteria), the presence of cyanophages, the genetic constitution of the strain, and its level of laboratory domestication (Wilson et al., 1996, 78031; Ni and Zeng, 2016; Adomako et al., 2022; Grasso et al., 2022). Also, the abiotic factors include the physical conditions of the culture (e.g., light intensity, photoperiod, and temperature) and the chemical composition of the culture medium (e.g., nitrogen source, salinity, N:P ratio, and CO_2_ levels) (Pathania and Srivastava, 2021). Consequently, the recorded proximate composition for the unaxenic cyanobacterium is relative and could change significantly. In other words, by obtaining an axenic strain of the cyanobacterium and changing the physicochemical conditions of the culture, the production of these main biomolecules could be modified. Nevertheless, regardless of using an unaxenic or axenic strain, the raw cyanobacterial biomass could be used to produce protein-enriched foods, biofuels (e.g., biodiesel and bioethanol), and several useful bioproducts through a biorefinery approach (Sivaramakrishnan et al., 2022; Wang et al., 2022).

The unaxenic cyanobacterium showed some typical cyanobacterial pigments with significant differences in their contents (Figure 3). All-trans-β-carotene was the main pigment (∼61 mg g^−1^ of fresh cyanobacterial biomass weight [fcbw]). The second more abundant pigment was total phycocyanin (17.95 ± 1.10 mg g^−1^ of fcbw) with c-phycocyanin (6.79 ± 0.43 mg g^−1^ of fcbw) and allophycocyanin (11.17 ± 0.68 mg g^−1^ of fcbw). The third more abundant pigment was chlorophyll a with a content of 7.62 ± 0.08 mg g^−1^ of fcbw. Finally, the pigment with the lowest content was lutein with a value of <1 mg g^−1^ of fcbw.

The metabolic capability of *Synechococcus* sp. UCP002 to produce the majority of these pigments is in conformity to its genetic information. The genome has all the genes coding the enzymes involved in the biosynthesis of all-trans-β-carotene and other carotenoids that were not quantified in this research such as lycopene, zeaxanthin, and astaxanthin (Supplementary Figure S5). Also, the genome has the genes coding the phycocyanins (*cpc*A, *cpc*B, *cpc*C, *cpc*D, *cpc*E, *cpc*F, and *cpc*G) and allophycocyanins (*apc*A, *apc*B, *apc*C, *apc*D, *apc*E, and *apc*F) and all the genes coding proteins involved in the assembly of the supramolecular complex called the phycobilisome. The phycobilisome is an efficient and versatile light-harvesting system that serves as a photosynthetic antenna in cyanobacteria (Watanabe and Ikeuchi, 2013; Adir et al., 2020; Grébert et al., 2022).

The genes coding proteins for assembly of the phycobilisome are clustered in two regions of 1.5 kbp (135,435–136,974) and 6.5 kbp (2,859,945–2,866,344) in the genome of *Synechococcus* sp. UCP002. This pattern of gene organization is common in most cyanobacteria of the *Synechococcus* genera, a first small cluster group of allophycocyanin core genes (*apc*E-A-B-C), while another core gene is formed by *apc*D and *apc*F. However, the phycobilisome rod genes are situated in larger clusters (from 6.5 to 28.5 Kbp), whose size depends on the complexity of the rod structure (Six et al., 2007; Grébert et al., 2022).

The genome of *Synechococcus* sp. UCP002 also harbors most of the genes coding the enzymes responsible for the biosynthesis of chlorophyll a (Supplementary Figure S6). However, the genes coding the enzymes for the biosynthesis of lutein, using lycopene as a metabolic precursor, were lacking in the genome. The missing genes coding the enzymes for the biosynthesis of lutein are typical in cyanobacteria of the *Synechococcus* genera (Sarnaik et al., 2018). Consequently, lutein identified in the unaxenic cyanobacterial biomass could be a result of its biosynthesis by the associated bacteria.

Additionally, the cyanobacterium strain showed the ability to produce phenolic compounds. The total phenolic content (TPC) recorded was 2.36 ± 0.06 mg GAE g^−1^ of cyanobacterial biomass dry weight. This TPC value is in the range of values reported for TPC in cyanobacteria. A similar low TPC is recorded in strains of the cyanobacterial genera *Arthrospira* (Cobos et al., 2020). Also, Li *et al.* reported that *Synechococcus* sp. FACHB 283 has a TPC of 10.56 ± 0.11 mg GAE g^−1^ of freeze-dried biomass weight (Li et al., 2007). In other cyanobacterial genera, such as *Aulosira*, *Anabaena*, *Aphanizomenon*, *Calothrix*, *Oscillatoria*, and *Synechocystis*, the TPC fluctuates from 22.17 to 290.23 mg GAE g^−1^ of fresh biomass weight (Singh et al., 2017; Senousy et al., 2020). Additionally, Del Mondo et al. recorded in 14 cyanobacterial genera (e.g., *Anabaena*, *Chroococcus*, *Fischerella*, *Plectonema*, and *Tolypothrix*) a TPC value from 1.0 to 60.53 mg GAE g^−1^ of cbdw (Del Mondo et al., 2021). Among the most common phenolic compounds identified in cyanobacteria are caffeic acid, chlorogenic acid, ferulic acid, gallic acid, protocatechuic acid, trans-cinnamic acid, *p*-coumaric acid, and vanillic acids (Babaoğlu Aydaş et al., 2013; Jerez-Martel et al., 2017; Singh et al., 2017; Del Mondo et al., 2021).

The notable differences in TPC and the class of phenolic acid biosynthesized by the different genera, species, and strains of cyanobacteria can be attributed to some factors. For example, variations in the culture conditions (e.g., light intensity, photoperiod, and the composition of the culture medium) affect noticeably the production of phenolic compounds (El-Baky et al., 2009; Blagojević et al., 2018). Also, a key factor for the biosynthesis of diverse phenolic compounds is the metabolic capabilities of the cyanobacteria. These metabolic capabilities ultimately depend on the genes coding the biosynthetic enzymes that harbor the cyanobacteria genomes. According to functional annotations using KAAS, the genome of *Synechococcus* sp. UCP002 has all the genes coding the enzymes of the shikimate pathway (Supplementary Figure S7). The shikimate pathway is the main aromatic biosynthetic pathway (Bentley, 1990; Mir et al., 2015). This metabolic pathway converts the metabolites erythrose-4-P and phosphoenolpyruvate to chorismate. Chorismate is the last common precursor for the biosynthesis of the three aromatic amino acids phenylalanine, tyrosine, and tryptophan (Herrmann, 1995). Furthermore, phenylalanine is the common metabolic precursor to biosynthesize multiple phenolic compounds through the phenylpropanoid biosynthetic pathway (Ferrer et al., 2008; Vogt, 2010).

However, it is intriguing that although the cyanobacterium produces phenolic compounds, not all the genes coding the 29 core enzymes of the phenylpropanoid/flavonoid biosynthetic pathway were found in the genome of *Synechococcus* sp. UCP002 (Supplementary Table S3). A total of 22 genes coding core enzymes were found in the genome but showed low pairwise identity with its corresponding orthologs (range from 21.4 to 39.3 %). However, seven genes coding the core enzymes were missing in the genome. These missing genes are those that code for phenylalanine ammonia-lyase (EC 4.3.1.24), tyrosine ammonia-lyase (EC 4.3.1.25), caffeic acid 3-O-methyltransferase (EC 2.1.1.68), and chalcone synthase (EC 2.3.1.74). This suggests that *Synechococcus* sp. UCP002 could use other unidentified enzymes that convert phenylalanine or tyrosine into the metabolic intermediaries cinnamic acid or *p*-coumaric acid, respectively. An analogous situation could be occurring with the other missing core enzymes of the phenylpropanoid/flavonoid biosynthetic pathway. Recently, it has been demonstrated that cyanobacteria display greater variability in this metabolic pathway, and several core enzymes (e.g., phenylalanine ammonia-lyase, chalcone synthase, and chalcone synthase) are missing in some analyzed cyanobacterial genera (e.g., *Fischerella*, *Mastigocoleus*, and *Cylindrospermum*) (Del Mondo et al., 2022).

#### 3.1.2 Fatty acid composition

The unaxenic cyanobacterium contained three groups of fatty acids, according to their level of saturation, namely, saturated fatty acids (SFAs), monounsaturated fatty acids (MUFAs), and polyunsaturated fatty acids (PUFAs) (Table 2). Palmitic acid (C16:0), palmitoleic acid (C16:1 n-7), and linoleic acid (C18:2 n-6) were the predominant ones in each fatty acid group. Comparing between groups, the content of MUFAs was the highest (ΣMUFA >52%), followed by the content of SFA (ΣSFA >36%), but the content of PUFAs was the lowest (ΣPUFA <0.5%). Also, five fatty acids were unknown (10.22% of total fatty acids). The pattern of fatty acid composition characterized by a high content of C16:0 and C16:1 n-7 fatty acids and low content of PUFAs is common in several cyanobacterial genera. These genera include *Anabaena* and *Nostoc* (Caudales and Wells, 1992; Guedes et al., 2011), *Cyanobacterium* (Sarsekeyeva et al., 2014), *Dermocarpa*, *Dermocarpella*, *Myxosarcina*, *Pleurocapsa*, *Xenococcus* (Caudales et al., 2000), *Aphanothece*, *Oscillatoria*, *Plectonema*, *Phormidium* (Oren et al., 1985), and *Synechococcus* (Murata et al., 1992; Takeyama et al., 1997; Yu et al., 2000; Santos-Merino et al., 2022). Consequently, these cyanobacteria do not produce the high-demanded polyunsaturated fatty acids EPA and DHA. These PUFAs are essential human nutrients biosynthesized for several eukaryotic microalgae (Nilsson et al., 2020; Hachicha et al., 2022; Jakhwal et al., 2022; Toumi et al., 2022).

**TABLE 2:** Fatty acid composition of the unaxenic cyanobacterium *Synechococcus* sp. UCP002 isolated from the Peruvian Amazon Basin region (mg.g^−1^ of cbdw).

Fatty acid	Mean	Standard deviation
Saturated fatty acid (SFA)		
C14:0 (myristic acid)	0.06	0.00
C16:0 (palmitic acid)	27.63	0.26
C18:0 (stearic acid)	0.42	0.01
Mono-unsaturated fatty acid (MUFA)		
C16:1 n-7 (palmitoleic acid)	34.51	0.31
C18:1 n-7 (vaccenic acid)	2.48	0.06
C18:1 n-9 (elaidic or oleic acid)	3.14	0.06
Polyunsaturated fatty acid (PUFA)		
C18:2 n-6 (linoleic acid)	0.29	0.02
Unknowns		
Unknown 1	5.56	0.02
Unknown 2	0.05	0.01
Unknown 3	0.28	0.01
Unknown 4	1.73	0.01
Unknown 5	0.18	0.00
ΣSFA	28.11 (36.83)	
ΣMUFA	40.13 (52.57)	
ΣPUFA	0.29 (0.38)	
Total fatty acids	76.33	

Legend: The concentration of fatty acids is expressed in mg/g of total lipids obtained from the unaxenic cyanobacterium dry biomass, with the percentage (%) of the total fatty acids in parentheses, and each value represents the mean ± SD of three experiments. ∑SFA is the sum of the contents of saturated fatty acids, ∑MUFA is the sum of the contents of mono-unsaturated fatty acids, and ∑PUFA is the sum of the contents of polyunsaturated fatty acids.

The inability of *Synechococcus* sp. UCP002 to biosynthesize long-chain PUFAs can be associated with its genetic makeup for *de novo* fatty acid biosynthesis. According to the functional annotation with KAAS and the metabolic pathways reconstructed, the genome of the cyanobacterium possesses the totality of genes coding the enzymes involved in *de novo* fatty acid biosynthesis (Supplementary Figure S8). The type of the fatty acid biosynthesis pathway identified is II FAS, which typically operates in prokaryotic microorganisms such as cyanobacteria (Mund et al., 2022). Recently, it has been demonstrated that the enzymes of the fatty acid biosynthesis pathway form a protein community called metabolon (Skalidis et al., 2022). This metabolon is generated as the final product of the SFAs palmitic acid (C16:0) and stearic acid (C18:0). Furthermore, both saturated fatty acids are chemically modified by the sequential action of desaturases and elongases. Consequently, depending on the gene pool contained in the genome that codes desaturases and elongases, the cyanobacterium will have the metabolic competence to biosynthesize or not a group of short-chain and/or long-chain PUFAs (Ratledge, 2004; Poole et al., 2020; Santos-Merino et al., 2022).

In this context and taking into account the modes of fatty acid desaturation, the cyanobacterium *Synechococcus* sp. UCP002 belongs to groups 1 and 2, such as was previously established for this cyanobacterium genus (Murata et al., 1992). According to this, the genes coding elongases were missing in the genome of the cyanobacterium. However, in the genome were identified two genes coding desaturases, the first coding one delta-9 desaturase and the second coding one delta-12 desaturase. Together, these results suggest that the hydrocarbon chain length and the pattern of desaturation (desaturations at the Δ^9^ and Δ^12^ positions) of the fatty acids biosynthesized by *Synechococcus* sp. UCP002 were determined by the content of genetic information in its genome.

#### 3.1.3 Amino acid composition

The unaxenic cyanobacterium strain contained 20 amino acids commonly found in proteins. In agreement with its nutritional value for human nutrition, it had both essential amino acids (EAA) and non-essential amino acids (NEAA). The content of both classes of amino acids in the cyanobacterial biomass was 45.62 and 54.38%, respectively (Table 3). Leucine was the main EAA, but histidine showed the lowest value (<10 mg g^−1^ of cbdw). In the NEAA group, glutamic acid (Glx) was more abundant and proline the scarcest (∼24 mg g^−1^ of cbdw). Consequently, the *Synechococcus* sp. UCP002 biomass could be a good source of essential amino acids for human and animal nutrition. Essential amino acids are a common component in proteins derived from the cyanobacterial and microalgal biomass (Kay, 1991; Hempel et al., 2012; Molino et al., 2018; Camacho et al., 2019; Cobos et al., 2020).

**TABLE 3:** Amino acid composition of the unaxenic cyanobacterium *Synechococcus* sp. UCP002 isolated from the Peruvian Amazon Basin region (mg.g^−1^ of cbdw).

Amino acid	Mean	Standard deviation
Essential amino acid (EAA)		
Valine	35.06	0.37
Threonine	33.04	0.45
Leucine	54.72	0.95
Isoleucine	33.03	0.40
Methionine + cysteine	10.44	0.69
Lysine	28.18	1.25
Histidine	9.32	0.16
Phenylalanine	30.94	0.45
Tyrosine	27.88	0.31
ΣEAA (%)	262.61 (45.62)	
Non-essential amino acid (NEAA)		
Glycine	31.03	0.43
Alanine	52.81	0.85
Serine	30.99	0.32
Proline	24.28	0.33
Arginine	41.69	0.95
Aspartic acid (Asx)	57.44	1.08
Glutamic acid (Glx)	74.74	1.12
ΣNEAA (%)	312.98 (54.38)	
Total AA	575.59	

Legend: The quantity of amino acids is expressed in mg.g^−1^ of cyanobacterial biomass dry weight, with percentage (%) of amino acid content in parentheses, and each value represents the mean ± SD of three experiments. ΣEAA is the sum of the essential amino acids, and ΣNEAA is the sum of the non-essential amino acids.

The capability to biosynthesize the 20 amino acids by *Synechococcus* sp. UCP002 was supported by its genetic information. First, the genome harbors the genes coding specific permeases for ammonium assimilation and the involved enzymes to incorporate the ammonium ions into carbon skeletons (i.e., glutamine synthetase and glutamate synthase) (Muro-Pastor and Florencio, 2003; Muro-Pastor et al., 2005). Second, the genome possesses the *nir*A-*nrt*ABCD-*nar*B operon (Supplementary Figure S9) for nitrate and nitrite assimilation (Omata et al., 1993). This operon is actively transcribed by nitrate and nitrite when the formation of glutamine decreases (Kikuchi et al., 1996). In *Synechococcus*, the best-characterized transcriptional activator of the *nir*A-*nrt*ABCD-*nar*B operon is codified by the gene *ntc*A (Vega-Palas et al., 1992; Luque et al., 1994). Finally, the genome has the complete set of genes coding the enzymes for the biosynthesis of all amino acids.

For example, the biosynthesis of the aromatic family of amino acids (i.e., phenylalanine, tyrosine, and tryptophan) begins with the condensation of d-erythrose 4-phosphate and phosphoenolpyruvate. This condensation reaction produces inorganic phosphate and 3-deoxy-d-arabino-heptulosonate-7-phosphate (DAHP). The biochemical reaction is catalyzed by the enzyme phospho-2-keto-3-deoxyheptonate aldolase, also called DAHP synthase (EC 2.5.1.54). This enzyme catalyzes the first committed step of the shikimate pathway, which produces chorismate by the consecutive catalytic activity of several enzymes (Riccardi et al., 1989) (Supplementary Figure S7). DHAP synthase is the regulatory enzyme, and its feedback inhibition is by metabolic intermediates (i.e., phenylpyruvate, prephenate, and chorismate) and by aromatic end products (i.e., phenylalanine, tyrosine, and tryptophan). This regulatory mechanism by feedback inhibition is common in several genera of cyanobacteria such as *Chlorogleopsis*, *Fischerella*, *Lyngbya*, *Synechococcus*, *Synechocystis*, *Oscillatoria*, and *Plectonema* (Hall et al., 1982). Chorismate produced in the shikimate pathway is a fundamental metabolic branch point to biosynthesize the three aromatic amino acids by the consecutive action of several enzymes (Riccardi et al., 1989).

## 3 Conclusion

The cyanobacterium *Synechococcus* sp. UCP002 shows the potential to be biotechnologically exploited for the following reasons: 1) owing to their genetic makeup, the cyanobacterium can biosynthesize biochemicals potentially useful for human and animal nutrition, 2) some of the main biomolecules produced by the cyanobacterium (i.e., lipids and carbohydrates) could be used as raw materials to produce biofuels, and 3) the genomic resources of the novel cyanobacterium strain could be used in the field of synthetic biology as a new source of known genes with genetic variations.

## Data Availability

The datasets presented in this study can be found in online repositories. The names of the repository/repositories and accession number(s) can be found in the article/Supplementary Material.

## References

[B1] Abalde J. Betancourt L. Torres E. Cid A. Barwell C. (1998). Purification and characterization of phycocyanin from the marine cyanobacterium Synechococcus sp. IO9201. Plant Sci. 136, 109–120. 10.1016/S0168-9452(98)00113-7

[B2] Adir N. Bar-Zvi S. Harris D. (2020). The amazing phycobilisome. Biochim. Biophys. Acta. Bioenerg. 1861, 148047. 10.1016/j.bbabio.2019.07.002 31306623

[B3] Adomako M. Ernst D. Simkovsky R. Chao Y.-Y. Wang J. Fang M. (2022). Comparative genomics of Synechococcus elongatus explains the phenotypic diversity of the strains. mBio 13, e0086222–22. 10.1128/mbio.00862-22 35475644 PMC9239245

[B4] Al-Haj L. Lui Y. T. Abed R. M. M. Gomaa M. A. Purton S. (2016). Cyanobacteria as chassis for industrial biotechnology: Progress and prospects. Life 6, 42. 10.3390/life6040042 27916886 PMC5198077

[B5] AlgaeBase (2022). Listing the World’s algae. Available at: https://www.algaebase.org/(Accessed September 17, 2022).

[B6] Allen M. M. (1968). Simple conditions for growth of unicellular blue-green algae on plates. J. Phycol. 4, 1–4. 10.1111/j.1529-8817.1968.tb04667.x 27067764

[B7] Allen M. M. Stanier R. Y. (1968). Growth and division of some unicellular blue-green algae. J. Gen. Microbiol. 51, 199–202. 10.1099/00221287-51-2-199 5652095

[B8] Alneberg J. Bjarnason B. S. de Bruijn I. Schirmer M. Quick J. Ijaz U. Z. (2014). Binning metagenomic contigs by coverage and composition. Nat. Methods 11, 1144–1146. 10.1038/nmeth.3103 25218180

[B9] Andrews S. (2010). FastQC A quality control tool for high throughput sequence data. Available at: https://www.bioinformatics.babraham.ac.uk/projects/fastqc/(Accessed April 19, 2020).

[B10] AOAC (1990). Official methods of analysis of the association of official analytical chemists (AOAC). 15th Edition. Washington, DC: AOAC International.

[B11] Arkin A. P. Cottingham R. W. Henry C. S. Harris N. L. Stevens R. L. Maslov S. (2018). KBase: The United States department of energy systems biology knowledgebase. Nat. Biotechnol. 36, 566–569. 10.1038/nbt.4163 29979655 PMC6870991

[B12] Babaoğlu Aydaş S. Ozturk S. Aslım B. (2013). Phenylalanine ammonia lyase (PAL) enzyme activity and antioxidant properties of some cyanobacteria isolates. Food Chem. 136, 164–169. 10.1016/j.foodchem.2012.07.119 23017408

[B13] Badr O. A. M. El-Shawaf I. I. S. El-Garhy H. A. S. Moustafa M. M. A. (2018). Isolation and molecular identification of two novel cyanobacterial isolates obtained from a stressed aquatic system. Gene Rep. 13, 110–114. 10.1016/j.genrep.2018.09.005

[B14] Bankevich A. Nurk S. Antipov D. Gurevich A. A. Dvorkin M. Kulikov A. S. (2012). SPAdes: A new genome assembly algorithm and its applications to single-cell sequencing. J. Comput. Biol. 19, 455–477. 10.1089/cmb.2012.0021 22506599 PMC3342519

[B15] Barrangou R. Fremaux C. Deveau H. Richards M. Boyaval P. Moineau S. (2007). CRISPR provides acquired resistance against viruses in prokaryotes. Science 315, 1709–1712. 10.1126/science.1138140 17379808

[B16] Bennett A. Bogorad L. (1973). Complementary chromatic adaptation in a filamentous blue-green alga. J. Cell Biol. 58, 419–435. 10.1083/jcb.58.2.419 4199659 PMC2109051

[B17] Bentley R. (1990). The shikimate pathway--a metabolic tree with many branches. Crit. Rev. Biochem. Mol. Biol. 25, 307–384. 10.3109/10409239009090615 2279393

[B18] Benzerara K. Skouri-Panet F. Li J. Férard C. Gugger M. Laurent T. (2014). Intracellular Ca-carbonate biomineralization is widespread in cyanobacteria. Proc. Natl. Acad. Sci. U. S. A. 111, 10933–10938. 10.1073/pnas.1403510111 25009182 PMC4121779

[B19] Blagojević D. Babić O. Rašeta M. Šibul F. Janjušević L. Simeunović J. (2018). Antioxidant activity and phenolic profile in filamentous cyanobacteria: The impact of nitrogen. J. Appl. Phycol. 30, 2337–2346. 10.1007/s10811-018-1476-4

[B20] Bligh E. G. Dyer W. J. (1959). A rapid method of total lipid extraction and purification. Can. J. Biochem. Physiol. 37, 911–917. 10.1139/o59-099 13671378

[B21] Bolger A. M. Lohse M. Usadel B. (2014). Trimmomatic: A flexible trimmer for Illumina sequence data. Bioinforma. Oxf. Engl. 30, 2114–2120. 10.1093/bioinformatics/btu170 PMC4103590 24695404

[B22] Brettin T. Davis J. J. Disz T. Edwards R. A. Gerdes S. Olsen G. J. (2015). RASTtk: A modular and extensible implementation of the rast algorithm for building custom annotation pipelines and annotating batches of genomes. Sci. Rep. 5, 8365. 10.1038/srep08365 25666585 PMC4322359

[B23] Busenlehner L. S. Pennella M. A. Giedroc D. P. (2003). The SmtB/ArsR family of metalloregulatory transcriptional repressors: Structural insights into prokaryotic metal resistance. FEMS Microbiol. Rev. 27, 131–143. 10.1016/S0168-6445(03)00054-8 12829264

[B24] Cai F. Axen S. D. Kerfeld C. A. (2013). Evidence for the widespread distribution of CRISPR-Cas system in the Phylum Cyanobacteria. RNA Biol. 10, 687–693. 10.4161/rna.24571 23628889 PMC3737326

[B25] Camacho C. Coulouris G. Avagyan V. Ma N. Papadopoulos J. Bealer K. (2009). BLAST+: Architecture and applications. BMC Bioinforma. 10, 421. 10.1186/1471-2105-10-421 PMC2803857 20003500

[B26] Camacho F. Macedo A. Malcata F. (2019). Potential industrial applications and commercialization of microalgae in the functional food and feed industries: A short review. Mar. Drugs 17, E312. 10.3390/md17060312 PMC6628611 31141887

[B27] Caudales R. Wells J. M. Butterfield J. E. (2000). Cellular fatty acid composition of cyanobacteria assigned to subsection II, order Pleurocapsales. Int. J. Syst. Evol. Microbiol. 50, 1029–1034. 10.1099/00207713-50-3-1029 10843042

[B28] Caudales R. Wells J. M. Y. (1992). Differentiation of free-living Anabaena and Nostoc cyanobacteria on the basis of fatty acid composition. Int. J. Syst. Bacteriol. 42, 246–251. 10.1099/00207713-42-2-246 1581185

[B29] Cavet J. S. Borrelly G. P. M. Robinson N. J. (2003). Zn, Cu and Co in cyanobacteria: Selective control of metal availability. FEMS Microbiol. Rev. 27, 165–181. 10.1016/S0168-6445(03)00050-0 12829266

[B30] Chaiklahan R. Chirasuwan N. Bunnag B. (2012). Stability of phycocyanin extracted from Spirulina sp.: Influence of temperature, pH and preservatives. Process Biochem. 47, 659–664. 10.1016/j.procbio.2012.01.010

[B31] Chaumeil P.-A. Mussig A. J. Hugenholtz P. Parks D. H. (2020). GTDB-tk: A toolkit to classify genomes with the genome taxonomy database. Bioinformatics 36, btz848–1927. 10.1093/bioinformatics/btz848 PMC7703759 31730192

[B32] Cobos M. Paredes J. D. Maddox J. D. Vargas-Arana G. Flores L. Aguilar C. P. (2017). Isolation and characterization of native microalgae from the Peruvian amazon with potential for biodiesel production. Energies 10, 224. 10.3390/en10020224

[B33] Cobos M. Pérez S. Braga J. Vargas-Arana G. Flores L. Paredes J. D. (2020). Nutritional evaluation and human health-promoting potential of compounds biosynthesized by native microalgae from the Peruvian Amazon. World J. Microbiol. Biotechnol. 36, 121. 10.1007/s11274-020-02896-1 32681243

[B34] Cohen S. A. Michaud D. P. (1993). Synthesis of a fluorescent derivatizing reagent, 6-aminoquinolyl-N-hydroxysuccinimidyl carbamate, and its application for the analysis of hydrolysate amino acids via high-performance liquid chromatography. Anal. Biochem. 211, 279–287. 10.1006/abio.1993.1270 8317704

[B35] Consortium U. (2021). UniProt: The universal protein knowledgebase in 2021. Nucleic Acids Res. 49, D480–D489. 10.1093/nar/gkaa1100 33237286 PMC7778908

[B36] Couvin D. Bernheim A. Toffano-Nioche C. Touchon M. Michalik J. Néron B. (2018). CRISPRCasFinder, an update of CRISRFinder, includes a portable version, enhanced performance and integrates search for Cas proteins. Nucleic Acids Res. 46, W246–W251. 10.1093/nar/gky425 29790974 PMC6030898

[B37] De Wever A. Benzerara K. Coutaud M. Caumes G. Poinsot M. Skouri-Panet F. (2019). Evidence of high Ca uptake by cyanobacteria forming intracellular CaCO3 and impact on their growth. Geobiology 17, 676–690. 10.1111/gbi.12358 31347755

[B38] Del Mondo A. Sansone C. Brunet C. (2022). Insights into the biosynthesis pathway of phenolic compounds in microalgae. Comput. Struct. Biotechnol. J. 20, 1901–1913. 10.1016/j.csbj.2022.04.019 35521550 PMC9052079

[B39] Del Mondo A. Smerilli A. Ambrosino L. Albini A. Noonan D. M. Sansone C. (2021). Insights into phenolic compounds from microalgae: Structural variety and complex beneficial activities from health to nutraceutics. Crit. Rev. Biotechnol. 41, 155–171. 10.1080/07388551.2021.1874284 33530761

[B40] Dierckxsens N. Mardulyn P. Smits G. (2017). NOVOPlasty: De novo assembly of organelle genomes from whole genome data. Nucleic Acids Res. 45, e18. 10.1093/nar/gkw955 28204566 PMC5389512

[B41] DuBois M. Gilles K. A. Hamilton J. K. Rebers P. A. Smith F. (1956). Colorimetric method for determination of sugars and related substances. Anal. Chem. 28, 350–356. 10.1021/ac60111a017

[B42] Dvořák P. Poulíčková A. Hašler P. Belli M. Casamatta D. A. Papini A. (2015). Species concepts and speciation factors in cyanobacteria, with connection to the problems of diversity and classification. Biodivers. Conserv. 24, 739–757. 10.1007/s10531-015-0888-6

[B43] El-Baky H. H. A. Baz F. E. El-Baroty G. S. (2009). Production of phenolic compounds from Spirulina maxima microalgae and its protective effects. Afr. J. Biotechnol. 8, 133. 10.4314/ajb.v8i24.68794

[B44] Ernst A. (1991). Cyanobacterial picoplankton from Lake Constance. I. Isolation by fluorescence characteristics. J. Plankton Res. 13, 1307–1312. 10.1093/plankt/13.6.1307

[B45] Ferrer J.-L. Austin M. B. Stewart C. Noel J. P. (2008). Structure and function of enzymes involved in the biosynthesis of phenylpropanoids. Plant Physiol. biochem. 46, 356–370. 10.1016/j.plaphy.2007.12.009 18272377 PMC2860624

[B46] Freitas T. A. K. Li P.-E. Scholz M. B. Chain P. S. G. (2015). Accurate read-based metagenome characterization using a hierarchical suite of unique signatures. Nucleic Acids Res. 43, e69. 10.1093/nar/gkv180 25765641 PMC4446416

[B47] Gordon G. C. Korosh T. C. Cameron J. C. Markley A. L. Begemann M. B. Pfleger B. F. (2016). CRISPR interference as a titratable, trans-acting regulatory tool for metabolic engineering in the cyanobacterium Synechococcus sp. strain PCC 7002. Metab. Eng. 38, 170–179. 10.1016/j.ymben.2016.07.007 27481676 PMC5107151

[B48] Grasso C. R. Pokrzywinski K. L. Waechter C. Rycroft T. Zhang Y. Aligata A. (2022). A review of cyanophage–host relationships: Highlighting cyanophages as a potential cyanobacteria control strategy. Toxins 14, 385. 10.3390/toxins14060385 35737046 PMC9229316

[B49] Grébert T. Garczarek L. Daubin V. Humily F. Marie D. Ratin M. (2022). Diversity and evolution of pigment types in marine Synechococcus cyanobacteria. Genome Biol. Evol. 14, evac035. 10.1093/gbe/evac035 35276007 PMC8995045

[B50] Grewe C. B. Pulz O. (2012). “The biotechnology of cyanobacteria,” in Ecology of cyanobacteria II: Their diversity in space and time. Editor Whitton B. A. (Dordrecht: Springer Netherlands), 707–739. 10.1007/978-94-007-3855-3_26

[B51] Guedes A. C. Amaro H. M. Barbosa C. R. Pereira R. D. Malcata F. X. (2011). Fatty acid composition of several wild microalgae and cyanobacteria, with a focus on eicosapentaenoic, docosahexaenoic and α-linolenic acids for eventual dietary uses. Food Res. Int. 44, 2721–2729. 10.1016/j.foodres.2011.05.020

[B52] Guiry M. D. (2012). How many species of algae are there? J. Phycol. 48, 1057–1063. 10.1111/j.1529-8817.2012.01222.x 27011267

[B53] Gupta A. Sainis J. K. (2010). Isolation of C-phycocyanin from Synechococcus sp., (anacystis nidulans BD1). J. Appl. Phycol. 22, 231–233. 10.1007/s10811-009-9449-2

[B54] Gurevich A. Saveliev V. Vyahhi N. Tesler G. (2013). Quast: Quality assessment tool for genome assemblies. Bioinformatics 29, 1072–1075. 10.1093/bioinformatics/btt086 23422339 PMC3624806

[B55] Hachicha R. Elleuch F. Ben Hlima H. Dubessay P. de Baynast H. Delattre C. (2022). Biomolecules from microalgae and cyanobacteria: Applications and market survey. Appl. Sci. 12, 1924. 10.3390/app12041924

[B56] Hall G. C. Flick M. B. Gherna R. L. Jensen R. A. (1982). Biochemical diversity for biosynthesis of aromatic amino acids among the cyanobacteria. J. Bacteriol. 149, 65–78. 10.1128/jb.149.1.65-78.1982 6119309 PMC216593

[B57] Hartree E. F. (1972). Determination of protein: A modification of the lowry method that gives a linear photometric response. Anal. Biochem. 48, 422–427. 10.1016/0003-2697(72)90094-2 4115981

[B58] Hempel N. Petrick I. Behrendt F. (2012). Biomass productivity and productivity of fatty acids and amino acids of microalgae strains as key characteristics of suitability for biodiesel production. J. Appl. Phycol. 24, 1407–1418. 10.1007/s10811-012-9795-3 23125481 PMC3478515

[B59] Herrmann K. M. (1995). The shikimate pathway as an entry to aromatic secondary metabolism. Plant Physiol. 107, 7–12. 10.1104/pp.107.1.7 7870841 PMC161158

[B60] Hirs C. H. Stein W. H. Moore S. (1954). The amino acid composition of ribonuclease. J. Biol. Chem. 211, 941–950. 10.1016/s0021-9258(18)71181-2 13221599

[B61] Hui C. Guo Y. Liu L. Yi J. (2021). Recent advances in bacterial biosensing and bioremediation of cadmium pollution: A mini-review. World J. Microbiol. Biotechnol. 38, 9. 10.1007/s11274-021-03198-w 34850291

[B62] Ichihara K. Fukubayashi Y. (2010). Preparation of fatty acid methyl esters for gas-liquid chromatography. J. Lipid Res. 51, 635–640. 10.1194/jlr.D001065 19759389 PMC2817593

[B63] Jaiswal D. Sengupta A. Sengupta S. Madhu S. Pakrasi H. B. Wangikar P. P. (2020). A novel cyanobacterium Synechococcus elongatus PCC 11802 has distinct genomic and metabolomic characteristics compared to its neighbor PCC 11801. Sci. Rep. 10, 191. 10.1038/s41598-019-57051-0 31932622 PMC6957532

[B64] Jaiswal D. Sengupta A. Sohoni S. Sengupta S. Phadnavis A. G. Pakrasi H. B. (2018). Genome features and biochemical characteristics of a robust, fast growing and naturally transformable cyanobacterium Synechococcus elongatus PCC 11801 isolated from India. Sci. Rep. 8, 16632. 10.1038/s41598-018-34872-z 30413737 PMC6226537

[B65] Jakhwal P. Kumar Biswas J. Tiwari A. Kwon E. E. Bhatnagar A. (2022). Genetic and non-genetic tailoring of microalgae for the enhanced production of eicosapentaenoic acid (EPA) and docosahexaenoic acid (DHA) – a review. Bioresour. Technol. 344, 126250. 10.1016/j.biortech.2021.126250 34728356

[B66] Jalili V. Afgan E. Gu Q. Clements D. Blankenberg D. Goecks J. (2020). The Galaxy platform for accessible, reproducible and collaborative biomedical analyses: 2020 update. Nucleic Acids Res. 48, W395–W402. 10.1093/nar/gkaa434 32479607 PMC7319590

[B67] Jasser I. Panou M. Khomutovska N. Sandzewicz M. Panteris E. Niyatbekov T. (2022). Cyanobacteria in hot pursuit: Characterization of cyanobacteria strains, including novel taxa, isolated from geothermal habitats from different ecoregions of the world. Mol. Phylogenet. Evol. 170, 107454. 10.1016/j.ympev.2022.107454 35341965

[B68] Jerez-Martel I. García-Poza S. Rodríguez-Martel G. Rico M. Afonso-Olivares C. Gómez-Pinchetti J. L. (2017). Phenolic profile and antioxidant activity of crude extracts from microalgae and cyanobacteria strains. J. Food Qual. 2017, 1–8. 10.1155/2017/2924508

[B69] Jian H. Xu G. Yi Y. Hao Y. Wang Y. Xiong L. (2021). The origin and impeded dissemination of the DNA phosphorothioation system in prokaryotes. Nat. Commun. 12, 6382. 10.1038/s41467-021-26636-7 34737280 PMC8569181

[B70] Kang D. D. Li F. Kirton E. Thomas A. Egan R. An H. (2019). MetaBAT 2: An adaptive binning algorithm for robust and efficient genome reconstruction from metagenome assemblies. PeerJ 7, e7359. 10.7717/peerj.7359 31388474 PMC6662567

[B71] Kauff F. Büdel B. (2011). “Phylogeny of cyanobacteria: An overview,” in *Progress in botany 72* progress in botany. Editors Lüttge U. E. Beyschlag W. Büdel B. Francis D. (Berlin, Heidelberg: Springer), 209–224. 10.1007/978-3-642-13145-5_8

[B72] Kay R. A. (1991). Microalgae as food and supplement. Crit. Rev. Food Sci. Nutr. 30, 555–573. 10.1080/10408399109527556 1741951

[B73] Kearse M. Moir R. Wilson A. Stones-Havas S. Cheung M. Sturrock S. (2012). Geneious Basic: An integrated and extendable desktop software platform for the organization and analysis of sequence data. Bioinformatics 28, 1647–1649. 10.1093/bioinformatics/bts199 22543367 PMC3371832

[B74] Kikuchi H. Aichi M. Suzuki I. Omato T. (1996). Positive regulation by nitrite of the nitrate assimilation operon in the cyanobacteria Synechococcus sp. strain PCC 7942 and Plectonema boryanum. J. Bacteriol. 178, 5822–5825. 10.1128/jb.178.19.5822-5825.1996 8824636 PMC178430

[B75] Kling J. D. Webb E. A. Hutchins D. A. (2022). Genome sequence of Synechococcus sp. strain LA31, isolated from a temperate estuary. Microbiol. Resour. Announc. 11, e0077521–21. 10.1128/mra.00775-21 35195452 PMC8928756

[B76] Kondo T. Mori T. Lebedeva N. V. Aoki S. Ishiura M. Golden S. S. (1997). Circadian rhythms in rapidly dividing cyanobacteria. Science 275, 224–227. 10.1126/science.275.5297.224 8985018

[B77] Koonin E. V. Makarova K. S. Wolf Y. I. (2017). Evolutionary genomics of defense systems in archaea and bacteria. Annu. Rev. Microbiol. 71, 233–261. 10.1146/annurev-micro-090816-093830 28657885 PMC5898197

[B78] Krawczyk P. S. Lipinski L. Dziembowski A. (2018). PlasFlow: Predicting plasmid sequences in metagenomic data using genome signatures. Nucleic Acids Res. 46, e35. 10.1093/nar/gkx1321 29346586 PMC5887522

[B79] Le S. Q. Gascuel O. (2008). An improved general amino acid replacement matrix. Mol. Biol. Evol. 25, 1307–1320. 10.1093/molbev/msn067 18367465

[B80] Lem N. W. Glick B. R. (1985). Biotechnological uses of cyanobacteria. Biotechnol. Adv. 3, 195–208. 10.1016/0734-9750(85)90291-5 14544049

[B81] Li D. Liu C.-M. Luo R. Sadakane K. Lam T.-W. (2015). Megahit: An ultra-fast single-node solution for large and complex metagenomics assembly via succinct de Bruijn graph. Bioinformatics 31, 1674–1676. 10.1093/bioinformatics/btv033 25609793

[B82] Li H.-B. Cheng K.-W. Wong C.-C. Fan K.-W. Chen F. Jiang Y. (2007). Evaluation of antioxidant capacity and total phenolic content of different fractions of selected microalgae. Food Chem. 102, 771–776. 10.1016/j.foodchem.2006.06.022

[B83] Lin W.-R. Tan S.-I. Hsiang C.-C. Sung P.-K. Ng I.-S. (2019). Challenges and opportunity of recent genome editing and multi-omics in cyanobacteria and microalgae for biorefinery. Bioresour. Technol. 291, 121932. 10.1016/j.biortech.2019.121932 31387837

[B84] Lu J. Chen F. Hodson R. E. (2001). Distribution, isolation, host specificity, and diversity of cyanophages infecting marine Synechococcus spp. in river estuaries. Appl. Environ. Microbiol. 67, 3285–3290. 10.1128/AEM.67.7.3285-3290.2001 11425754 PMC93013

[B85] Luque I. Flores E. Herrero A. (1994). Molecular mechanism for the operation of nitrogen control in cyanobacteria. EMBO J. 13, 2862–2869. 10.1002/j.1460-2075.1994.tb06580.x 8026471 PMC395167

[B86] Ma Z. Jacobsen F. E. Giedroc D. P. (2009). Coordination chemistry of bacterial metal transport and sensing. Chem. Rev. 109, 4644–4681. 10.1021/cr900077w 19788177 PMC2783614

[B87] Makarova K. S. Wolf Y. I. Koonin E. V. (2013). Comparative genomics of defense systems in archaea and bacteria. Nucleic Acids Res. 41, 4360–4377. 10.1093/nar/gkt157 23470997 PMC3632139

[B88] Marraffini L. A. Sontheimer E. J. (2010). CRISPR interference: RNA-directed adaptive immunity in bacteria and archaea. Nat. Rev. Genet. 11, 181–190. 10.1038/nrg2749 20125085 PMC2928866

[B89] Mastropetros S. G. Pispas K. Zagklis D. Ali S. S. Kornaros M. (2022). Biopolymers production from microalgae and cyanobacteria cultivated in wastewater: Recent advances. Biotechnol. Adv. 60, 107999. 10.1016/j.biotechadv.2022.107999 35667537

[B90] Mir R. Jallu S. Singh T. P. (2015). The shikimate pathway: Review of amino acid sequence, function and three-dimensional structures of the enzymes. Crit. Rev. Microbiol. 41, 172–189. 10.3109/1040841X.2013.813901 23919299

[B91] Molino A. Iovine A. Casella P. Mehariya S. Chianese S. Cerbone A. (2018). Microalgae characterization for consolidated and new application in human food, animal feed and nutraceuticals. Int. J. Environ. Res. Public Health 15, E2436. 10.3390/ijerph15112436 PMC6266511 30388801

[B92] Mori T. Binder B. Johnson C. H. (1996). Circadian gating of cell division in cyanobacteria growing with average doubling times of less than 24 hours. Proc. Natl. Acad. Sci. U. S. A. 93, 10183–10188. 10.1073/pnas.93.19.10183 8816773 PMC38358

[B93] Moriya Y. Itoh M. Okuda S. Yoshizawa A. C. Kanehisa M. (2007). Kaas: An automatic genome annotation and pathway reconstruction server. Nucleic Acids Res. 35, W182–W185. 10.1093/nar/gkm321 17526522 PMC1933193

[B94] Mund N. K. Liu Y. Chen S. (2022). Advances in metabolic engineering of cyanobacteria for production of biofuels. Fuel 322, 124117. 10.1016/j.fuel.2022.124117

[B95] Murata N. Wada H. Gombos Z. (1992). Modes of fatty-acid desaturation in cyanobacteria. Plant Cell Physiol. 33, 933–941. 10.1093/oxfordjournals.pcp.a078344

[B96] Muro-Pastor M. I. Florencio F. J. (2003). Regulation of ammonium assimilation in cyanobacteria. Plant Physiology Biochem. 41, 595–603. 10.1016/S0981-9428(03)00066-4

[B97] Muro-Pastor M. I. Reyes J. C. Florencio F. J. (2005). Ammonium assimilation in cyanobacteria. Photosynth. Res. 83, 135–150. 10.1007/s11120-004-2082-7 16143848

[B98] Ni T. Zeng Q. (2016). Diel infection of cyanobacteria by cyanophages. Front. Mar. Sci. 2. Available at: https://www.frontiersin.org/articles/10.3389/fmars.2015.00123 (Accessed September 15, 2022).

[B99] Nilsson A. K. Jiménez C. Wulff A. (2020). “Nutraceutical fatty acid production in marine microalgae and cyanobacteria,” in Nutraceutical fatty acids from oleaginous microalgae (New Jersey: John Wiley & Sons), 23–76. 10.1002/9781119631729.ch2

[B100] Nurk S. Meleshko D. Korobeynikov A. Pevzner P. A. (2017). metaSPAdes: a new versatile metagenomic assembler. Genome Res. 27, 824–834. 10.1101/gr.213959.116 28298430 PMC5411777

[B101] Omata T. Andriesse X. Hirano A. (1993). Identification and characterization of a gene cluster involved in nitrate transport in the cyanobacterium Synechococcus sp. PCC7942. Mol. Gen. Genet. 236, 193–202. 10.1007/BF00277112 8437564

[B102] Oren A. Fattom A. Padan E. Tietz A. (1985). Unsaturated fatty acid composition and biosynthesis in Oscillatoria limnetica and other cyanobacteria. Arch. Microbiol. 141, 138–142. 10.1007/BF00423274

[B103] Parks D. H. Chuvochina M. Rinke C. Mussig A. J. Chaumeil P.-A. Hugenholtz P. (2022). Gtdb: An ongoing census of bacterial and archaeal diversity through a phylogenetically consistent, rank normalized and complete genome-based taxonomy. Nucleic Acids Res. 50, D785–D794. 10.1093/nar/gkab776 34520557 PMC8728215

[B104] Parks D. H. Imelfort M. Skennerton C. T. Hugenholtz P. Tyson G. W. (2015). CheckM: Assessing the quality of microbial genomes recovered from isolates, single cells, and metagenomes. Genome Res. 25, 1043–1055. 10.1101/gr.186072.114 25977477 PMC4484387

[B105] Pathania R. Srivastava S. (2021). Synechococcus elongatus BDU 130192, an attractive cyanobacterium for feedstock applications: Response to culture conditions. Bioenerg. Res. 14, 954–963. 10.1007/s12155-020-10207-7

[B106] Pattharaprachayakul N. Lee M. Incharoensakdi A. Woo H. M. (2020). Current understanding of the cyanobacterial CRISPR-Cas systems and development of the synthetic CRISPR-Cas systems for cyanobacteria. Enzyme Microb. Technol. 140, 109619. 10.1016/j.enzmictec.2020.109619 32912679

[B107] Peng Y. Leung H. C. M. Yiu S. M. Chin F. Y. L. (2012). IDBA-UD: A de novo assembler for single-cell and metagenomic sequencing data with highly uneven depth. Bioinformatics 28, 1420–1428. 10.1093/bioinformatics/bts174 22495754

[B108] Pierpont C. L. Ohkubo S. Miyashita H. Miller S. R. (2022). Draft genome sequence of the cyanobacterium Synechococcus sp. strain Nb3U1. Microbiol. Resour. Announc. 11, e0025122–22. 10.1128/mra.00251-22 35438510 PMC9119104

[B109] Poole L. B. Parsonage D. Sergeant S. Miller L. R. Lee J. Furdui C. M. (2020). Acyl-lipid desaturases and Vipp1 cooperate in cyanobacteria to produce novel omega-3 PUFA-containing glycolipids. Biotechnol. Biofuels 13, 83. 10.1186/s13068-020-01719-7 32399061 PMC7203895

[B110] Prabha S. Vijay A. K. Paul R. R. George B. (2022). Cyanobacterial biorefinery: Towards economic feasibility through the maximum valorization of biomass. Sci. Total Environ. 814, 152795. 10.1016/j.scitotenv.2021.152795 34979226

[B111] Prihantini N. B. (2020). Morphological identification, isolation, and culturing of cyanobacteria derived from hot spring of Cisolok and Galunggung Mountain based on enrichment method. J. Phys. Conf. Ser. 1442, 012069. 10.1088/1742-6596/1442/1/012069

[B112] Purdy H. M. Pfleger B. F. Reed J. L. (2022). Introduction of NADH-dependent nitrate assimilation in Synechococcus sp. PCC 7002 improves photosynthetic production of 2-methyl-1-butanol and isobutanol. Metab. Eng. 69, 87–97. 10.1016/j.ymben.2021.11.003 34774761 PMC9026717

[B113] Racharaks R. Arnold W. Peccia J. (2021). Development of CRISPR-Cas9 knock-in tools for free fatty acid production using the fast-growing cyanobacterial strain Synechococcus elongatus UTEX 2973. J. Microbiol. Methods 189, 106315. 10.1016/j.mimet.2021.106315 34454980

[B114] Ratledge C. (2004). Fatty acid biosynthesis in microorganisms being used for Single Cell Oil production. Biochimie 86, 807–815. 10.1016/j.biochi.2004.09.017 15589690

[B115] Riccardi G. de Rossi E. Milano A. (1989). Amino acid biosynthesis and its regulation in cyanobacteria. Plant Sci. 64, 135–151. 10.1016/0168-9452(89)90018-6

[B116] Richmond A. Hu Q. (2013). Handbook of microalgal culture: Applied phycology and biotechnology. Second Edition. New Jersey: John Wiley & Sons.

[B117] Sambrook J. Russell D. W. (2006). The condensed protocols from molecular cloning: A laboratory manual. 1st edition. New York: Cold Spring Harbor Laboratory Press.

[B118] Santos-Merino M. Gutiérrez-Lanza R. Nogales J. García J. L. de la Cruz F. (2022). Synechococcus elongatus PCC 7942 as a platform for bioproduction of omega-3 fatty acids. Life 12, 810. 10.3390/life12060810 35743841 PMC9224711

[B119] Sarnaik A. Nambissan V. Pandit R. Lali A. (2018). Recombinant Synechococcus elongatus PCC 7942 for improved zeaxanthin production under natural light conditions. Algal Res. 36, 139–151. 10.1016/j.algal.2018.10.021

[B120] Sarsekeyeva F. K. Usserbaeva A. A. Zayadan B. K. Mironov K. S. Sidorov R. A. Kozlova A. Y. (2014). Isolation and characterization of a new cyanobacterial strain with a unique fatty acid composition. Adv. Microbiol. 04, 1033–1043. 10.4236/aim.2014.415114

[B121] Schwengers O. Jelonek L. Dieckmann M. A. Beyvers S. Blom J. Goesmann A. (2021). Bakta: Rapid and standardized annotation of bacterial genomes via alignment-free sequence identification. Microb. Genom. 7, 000685. 10.1099/mgen.0.000685 34739369 PMC8743544

[B122] Seemann T. (2014). Prokka: Rapid prokaryotic genome annotation. Bioinformatics 30, 2068–2069. 10.1093/bioinformatics/btu153 24642063

[B123] Selão T. T. (2022). Exploring cyanobacterial diversity for sustainable biotechnology. J. Exp. Bot. 73, 3057–3071. 10.1093/jxb/erac053 35467729

[B124] Senousy H. H. Abd Ellatif S. Ali S. (2020). Assessment of the antioxidant and anticancer potential of different isolated strains of cyanobacteria and microalgae from soil and agriculture drain water. Environ. Sci. Pollut. Res. Int. 27, 18463–18474. 10.1007/s11356-020-08332-z 32193737

[B125] Shaffer M. Borton M. A. McGivern B. B. Zayed A. A. La Rosa S. L. Solden L. M. (2020). DRAM for distilling microbial metabolism to automate the curation of microbiome function. Nucleic Acids Res. 48, 8883–8900. 10.1093/nar/gkaa621 32766782 PMC7498326

[B126] Sieber C. M. K. Probst A. J. Sharrar A. Thomas B. C. Hess M. Tringe S. G. (2018). Recovery of genomes from metagenomes via a dereplication, aggregation and scoring strategy. Nat. Microbiol. 3, 836–843. 10.1038/s41564-018-0171-1 29807988 PMC6786971

[B127] Simão F. A. Waterhouse R. M. Ioannidis P. Kriventseva E. V. Zdobnov E. M. (2015). BUSCO: Assessing genome assembly and annotation completeness with single-copy orthologs. Bioinformatics 31, 3210–3212. 10.1093/bioinformatics/btv351 26059717

[B128] Singh D. P. Prabha R. Verma S. Meena K. K. Yandigeri M. (2017). Antioxidant properties and polyphenolic content in terrestrial cyanobacteria. 3 Biotech. 7, 134. 10.1007/s13205-017-0786-6 PMC5462663 28593520

[B129] Singh P. Kumar D. (2022). Biomass and lipid production potential of cyanobacteria and microalgae isolated from the diverse habitats of Garhwal Himalaya, Uttarakhand, India. Biomass Bioenergy 162, 106469. 10.1016/j.biombioe.2022.106469

[B130] Singh S. Kate B. N. Banerjee U. C. (2005). Bioactive compounds from cyanobacteria and microalgae: An overview. Crit. Rev. Biotechnol. 25, 73–95. 10.1080/07388550500248498 16294828

[B131] Sivaramakrishnan R. Suresh S. Kanwal S. Ramadoss G. Ramprakash B. Incharoensakdi A. (2022). Microalgal biorefinery concepts’ developments for biofuel and bioproducts: Current perspective and bottlenecks. Int. J. Mol. Sci. 23, 2623. 10.3390/ijms23052623 35269768 PMC8910654

[B132] Six C. Thomas J.-C. Garczarek L. Ostrowski M. Dufresne A. Blot N. (2007). Diversity and evolution of phycobilisomes in marine Synechococcus spp.: A comparative genomics study. Genome Biol. 8, R259. 10.1186/gb-2007-8-12-r259 18062815 PMC2246261

[B133] Skalidis I. Kyrilis F. L. Tüting C. Hamdi F. Chojnowski G. Kastritis P. L. (2022). Cryo-EM and artificial intelligence visualize endogenous protein community members. Structure 30, 575–589.e6. 10.1016/j.str.2022.01.001 35093201

[B134] Sonani R. R. Patel S. Bhastana B. Jakharia K. Chaubey M. G. Singh N. K. (2017). Purification and antioxidant activity of phycocyanin from Synechococcus sp. R42DM isolated from industrially polluted site. Bioresour. Technol. 245, 325–331. 10.1016/j.biortech.2017.08.129 28898827

[B135] Sorek R. Kunin V. Hugenholtz P. (2008). CRISPR--a widespread system that provides acquired resistance against phages in bacteria and archaea. Nat. Rev. Microbiol. 6, 181–186. 10.1038/nrmicro1793 18157154

[B136] Sugita C. Ogata K. Shikata M. Jikuya H. Takano J. Furumichi M. (2007). Complete nucleotide sequence of the freshwater unicellular cyanobacterium Synechococcus elongatus PCC 6301 chromosome: Gene content and organization. Photosynth. Res. 93, 55–67. 10.1007/s11120-006-9122-4 17211581

[B137] Suttle C. A. Chan A. M. (1994). Dynamics and distribution of cyanophages and their effect on marine Synechococcus spp. Appl. Environ. Microbiol. 60, 3167–3174. 10.1128/aem.60.9.3167-3174.1994 16349372 PMC201785

[B138] Takeyama H. Takeda D. Yazawa K. Yamada A. Matsunaga T. (1997). Expression of the eicosapentaenoic acid synthesis gene cluster from Shewanella sp. in a transgenic marine cyanobacterium, Synechococcus sp. Microbiol. Read. 143, 2725–2731. 10.1099/00221287-143-8-2725 9274025

[B139] Tamura K. Stecher G. Kumar S. (2021). MEGA11: Molecular evolutionary genetics analysis version 11. Mol. Biol. Evol. 38, 3022–3027. 10.1093/molbev/msab120 33892491 PMC8233496

[B140] Tan L.-R. Cao Y.-Q. Li J.-W. Xia P.-F. Wang S.-G. (2022). Transcriptomics and metabolomics of engineered Synechococcus elongatus during photomixotrophic growth. Microb. Cell Fact. 21, 31. 10.1186/s12934-022-01760-1 35248031 PMC8897908

[B141] Tanizawa Y. Fujisawa T. Nakamura Y. (2018). Dfast: A flexible prokaryotic genome annotation pipeline for faster genome publication. Bioinformatics 34, 1037–1039. 10.1093/bioinformatics/btx713 29106469 PMC5860143

[B142] Thajuddin N. Subramanian G. (2005). Cyanobacterial biodiversity and potential applications in biotechnology. Curr. Sci. 89, 47–57.

[B143] Thilak T. S. Madhusoodanan P. V. Pradeep N. S. Prakashkumar R. (2020). Isolation and taxonomy of the blue-green algae (cyanobacteria), Nostoc and Anabaena in Kerala state, India. Acta Bot. Hung. 62, 163–174. 10.1556/034.62.2020.1-2.10

[B144] Toumi A. Politaeva N. Đurović S. Mukhametova L. Ilyashenko S. (2022). Obtaining DHA–EPA oil concentrates from the biomass of microalga chlorella sorokiniana. Resources 11, 20. 10.3390/resources11020020

[B145] Turner J. S. Glands P. D. Samson A. C. R. Robinson N. J. (1996). Zn 2+ -sensing by the cyanobacterial metallothionein repressor SmtB: Different motifs mediate metal-induced protein-DNA dissociation. Nucleic Acids Res. 24, 3714–3721. 10.1093/nar/24.19.3714 8871549 PMC146171

[B146] Udayan A. Arumugam M. Pandey A. (2017). “Chapter 4 - nutraceuticals from algae and cyanobacteria,” in Algal green chemistry (Amsterdam: Elsevier), 65–89. 10.1016/B978-0-444-63784-0.00004-7

[B147] Ungerer J. Lin P.-C. Chen H.-Y. Pakrasi H. B. (2018a). Adjustments to photosystem stoichiometry and electron transfer proteins are key to the remarkably fast growth of the cyanobacterium Synechococcus elongatus UTEX 2973. mBio 9, e02327–17. 10.1128/mBio.02327-17 29437923 PMC5801466

[B148] Ungerer J. Wendt K. E. Hendry J. I. Maranas C. D. Pakrasi H. B. (2018b). Comparative genomics reveals the molecular determinants of rapid growth of the cyanobacterium Synechococcus elongatus UTEX 2973. Proc. Natl. Acad. Sci. U. S. A. 115, E11761–E11770. 10.1073/pnas.1814912115 30409802 PMC6294925

[B149] Vega-Palas M. A. Flores E. Herrero A. (1992). NtcA, a global nitrogen regulator from the cyanobacterium Synechococcus that belongs to the Crp family of bacterial regulators. Mol. Microbiol. 6, 1853–1859. 10.1111/j.1365-2958.1992.tb01357.x 1630321

[B150] Velioglu Y. S. Mazza G. Gao L. Oomah B. D. (1998). Antioxidant activity and total phenolics in selected fruits, vegetables, and grain products. J. Agric. Food Chem. 46, 4113–4117. 10.1021/jf9801973

[B151] Vogt T. (2010). Phenylpropanoid biosynthesis. Mol. Plant 3, 2–20. 10.1093/mp/ssp106 20035037

[B152] Wang S. Mukhambet Y. Esakkimuthu S. Abomohra A. E.-F. (2022). Integrated microalgal biorefinery – routes, energy, economic and environmental perspectives. J. Clean. Prod. 348, 131245. 10.1016/j.jclepro.2022.131245

[B153] Watanabe M. Ikeuchi M. (2013). Phycobilisome: Architecture of a light-harvesting supercomplex. Photosynth. Res. 116, 265–276. 10.1007/s11120-013-9905-3 24081814

[B154] Wendt K. E. Walker P. Sengupta A. Ungerer J. Pakrasi H. B. (2022). Engineering natural competence into the fast-growing cyanobacterium Synechococcus elongatus strain UTEX 2973. Appl. Environ. Microbiol. 88, e0188221–21. 10.1128/AEM.01882-21 34705549 PMC8752150

[B155] Wick R. R. Judd L. M. Gorrie C. L. Holt K. E. (2017). Unicycler: Resolving bacterial genome assemblies from short and long sequencing reads. PLoS Comput. Biol. 13, e1005595. 10.1371/journal.pcbi.1005595 28594827 PMC5481147

[B156] Wilson W. H. Carr N. G. Mann N. H. (1996). The effect of phosphate status on the kinetics of cyanophage infection in the oceanic cyanobacterium Synechococcus sp. Wh78031. J. Phycol. 32, 506–516. 10.1111/j.0022-3646.1996.00506.x

[B157] Włodarczyk A. Selão T. T. Norling B. Nixon P. J. (2020). Newly discovered Synechococcus sp. PCC 11901 is a robust cyanobacterial strain for high biomass production. Commun. Biol. 3, 215. 10.1038/s42003-020-0910-8 32382027 PMC7205611

[B158] Wu M. Eisen J. A. (2008). A simple, fast, and accurate method of phylogenomic inference. Genome Biol. 9, R151. 10.1186/gb-2008-9-10-r151 18851752 PMC2760878

[B159] Wu Y.-W. Simmons B. A. Singer S. W. (2016). MaxBin 2.0: An automated binning algorithm to recover genomes from multiple metagenomic datasets. Bioinformatics 32, 605–607. 10.1093/bioinformatics/btv638 26515820

[B160] Yu J. Liberton M. Cliften P. F. Head R. D. Jacobs J. M. Smith R. D. (2015). Synechococcus elongatus UTEX 2973, a fast growing cyanobacterial chassis for biosynthesis using light and CO₂. Sci. Rep. 5, 8132. 10.1038/srep08132 25633131 PMC5389031

[B161] Yu R. Yamada A. Watanabe K. Yazawa K. Takeyama H. Matsunaga T. (2000). Production of eicosapentaenoic acid by a recombinant marine cyanobacterium, Synechococcus sp. Lipids 35, 1061–1064. 10.1007/s11745-000-0619-6 11104010

[B162] Zerbino D. R. (2010). Using the Velvet de novo Assembler for Short-Read Sequencing Technologies. Curr. Protoc. Bioinforma. 31, 11.5. 10.1002/0471250953.bi1105s31 PMC2952100 20836074

[B163] Zimin A. V. Marçais G. Puiu D. Roberts M. Salzberg S. L. Yorke J. A. (2013). The MaSuRCA genome assembler. Bioinformatics 29, 2669–2677. 10.1093/bioinformatics/btt476 23990416 PMC3799473

