# Supplementary figures and images for: Genomic analysis and biochemical profiling of an unaxenic strain of Synechococcus sp. isolated from the Peruvian Amazon Basin region
Source: Front Genet. 2022 Nov 9;13:973324. doi: 10.3389/fgene.2022.973324 (PMC9682099; doi:10.3389/fgene.2022.973324)

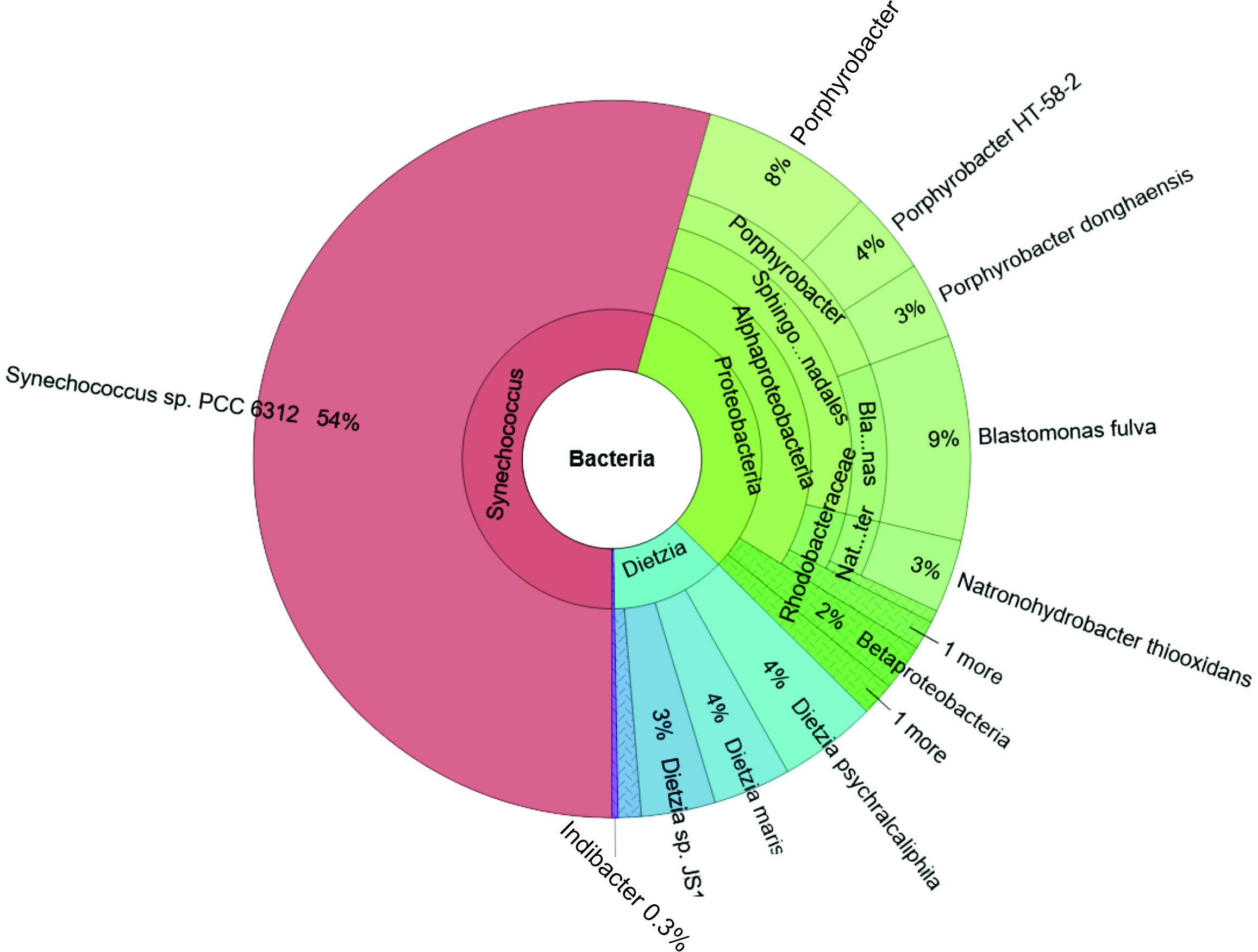

Supplement: Supplementary file 2 [file Image3.JPEG]

Coverage: 182x

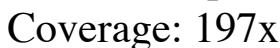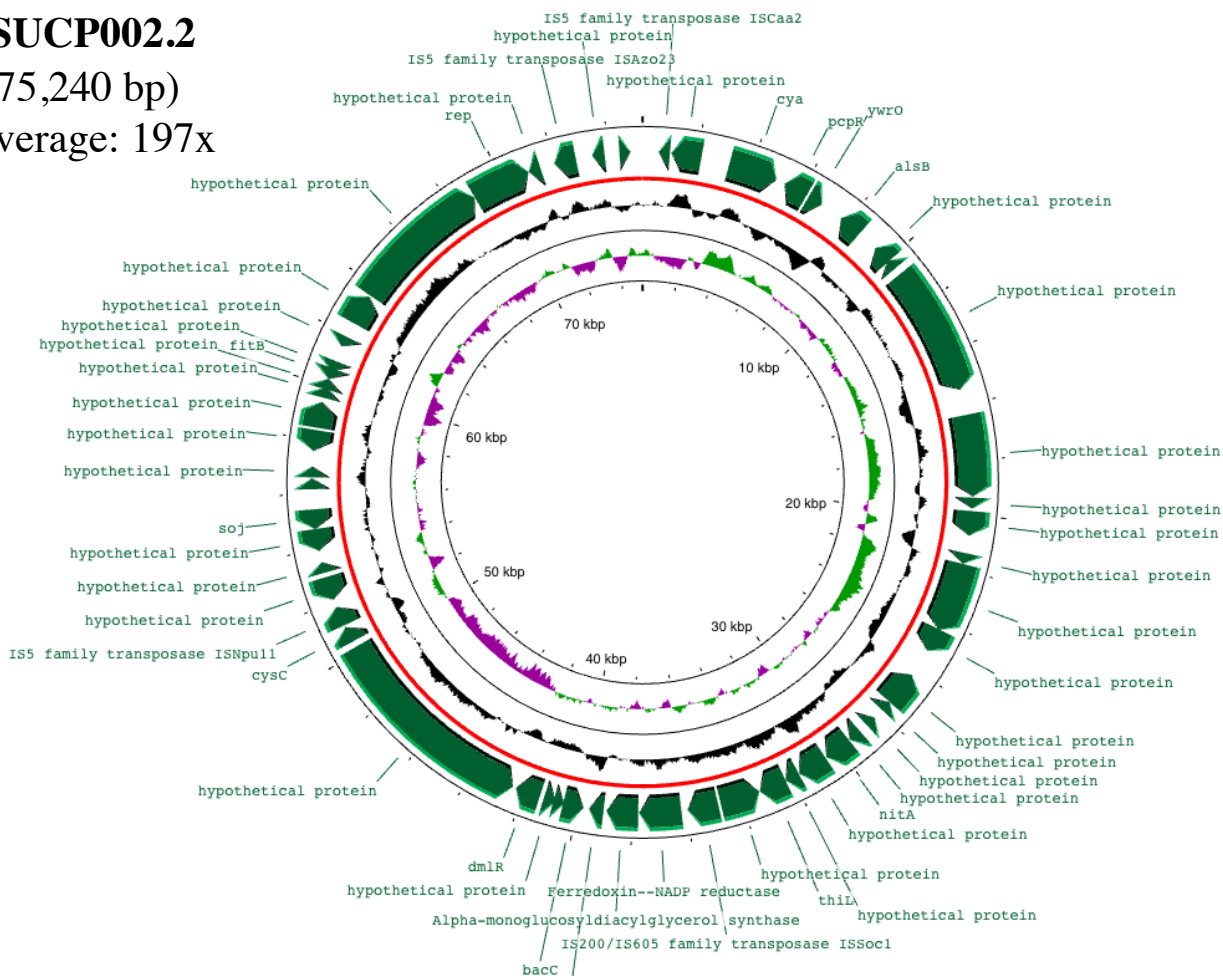

Coverage: 349x

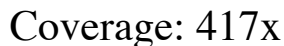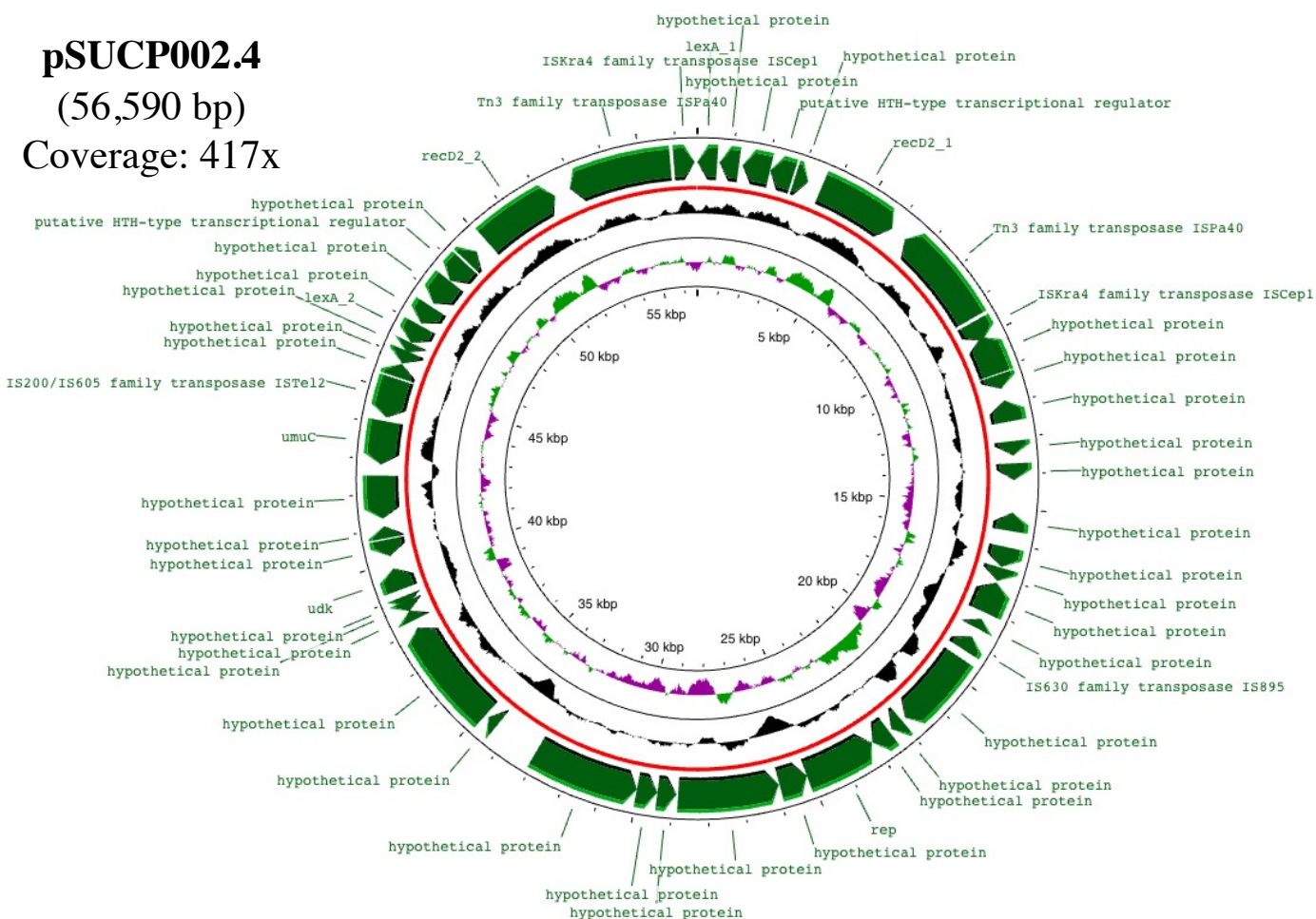

(24,438 bp)

Coverage: 238x

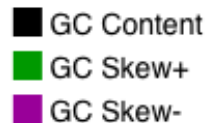

(200,030 bp)

Coverage: 215x

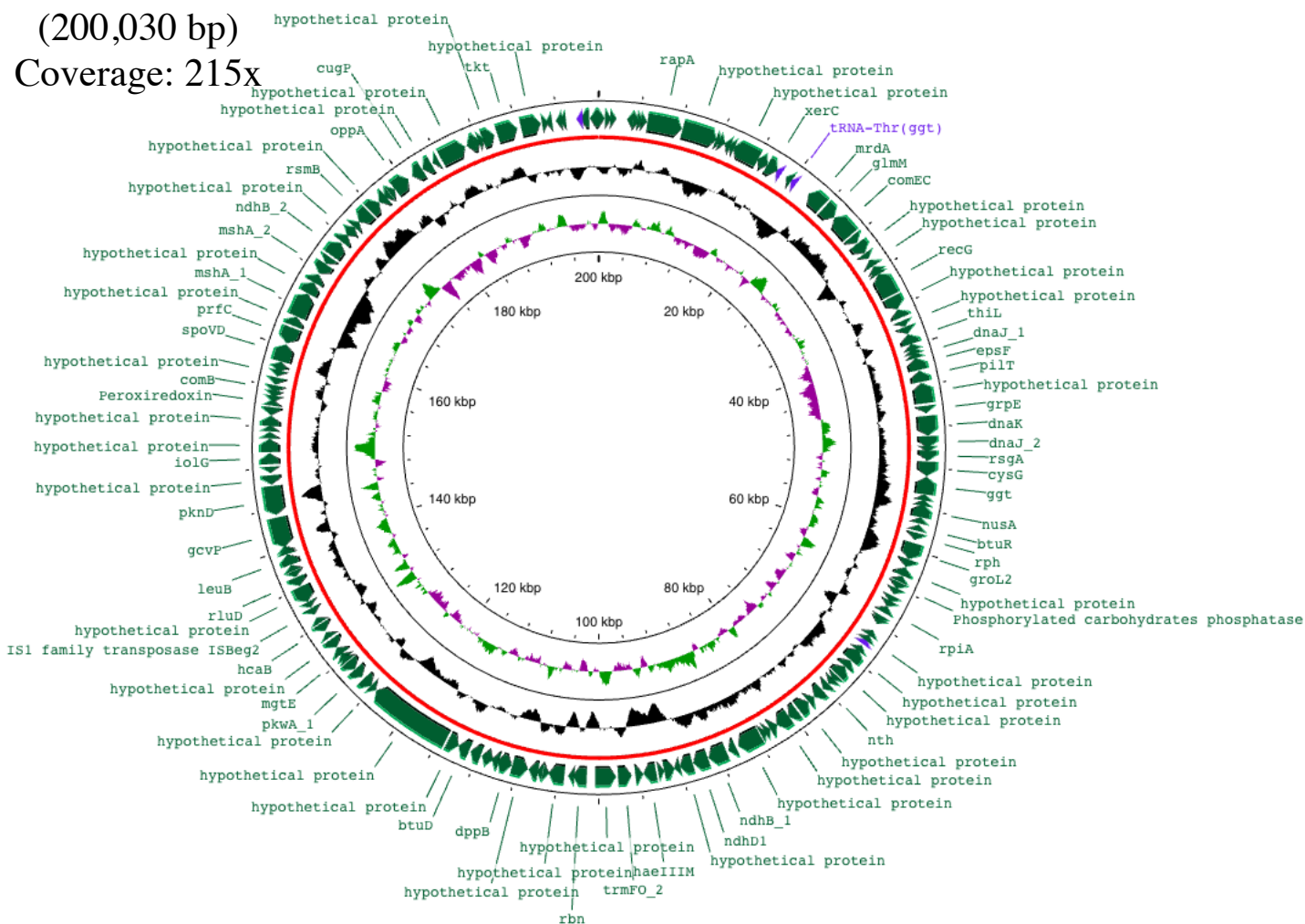

Supplement: Supplementary file 4 [file Image9.PDF]

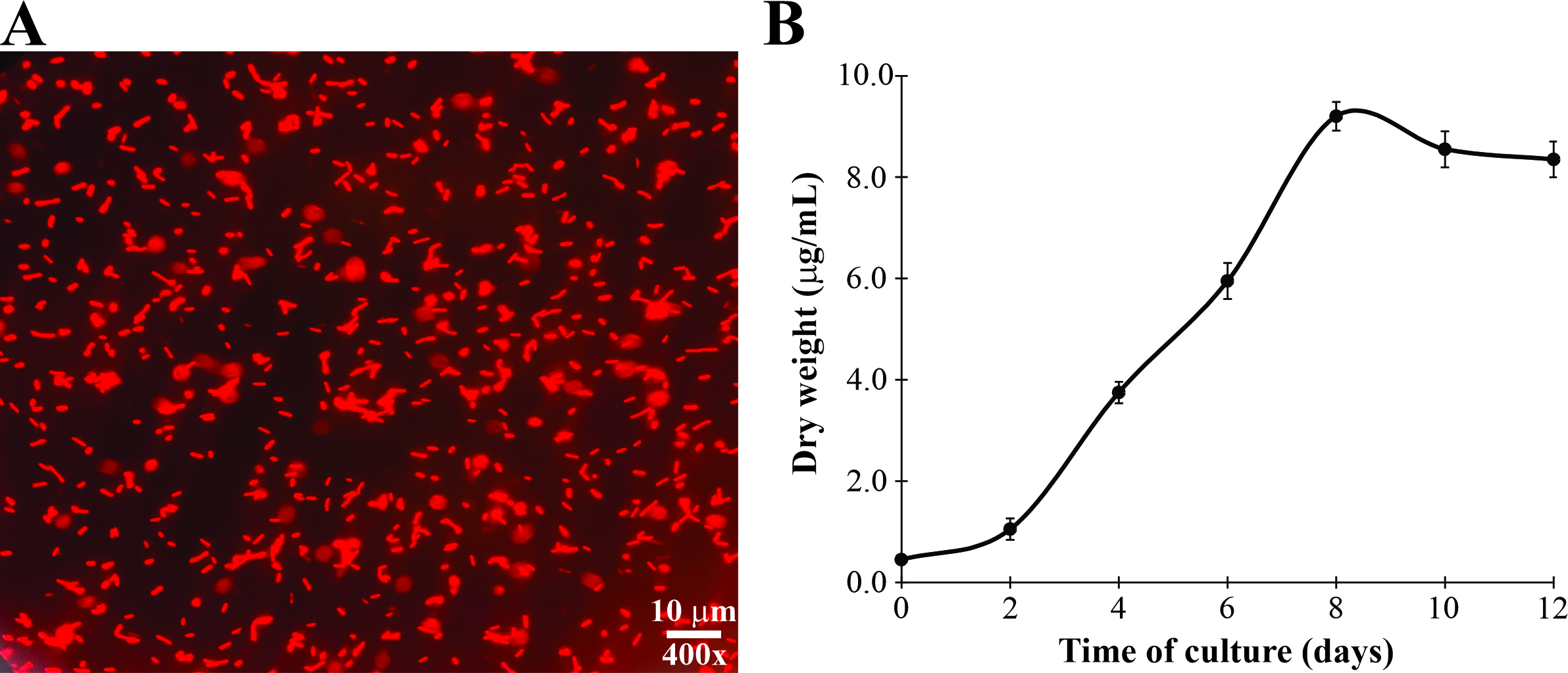

Supplement: Supplementary file 5 [file Image1.JPEG]

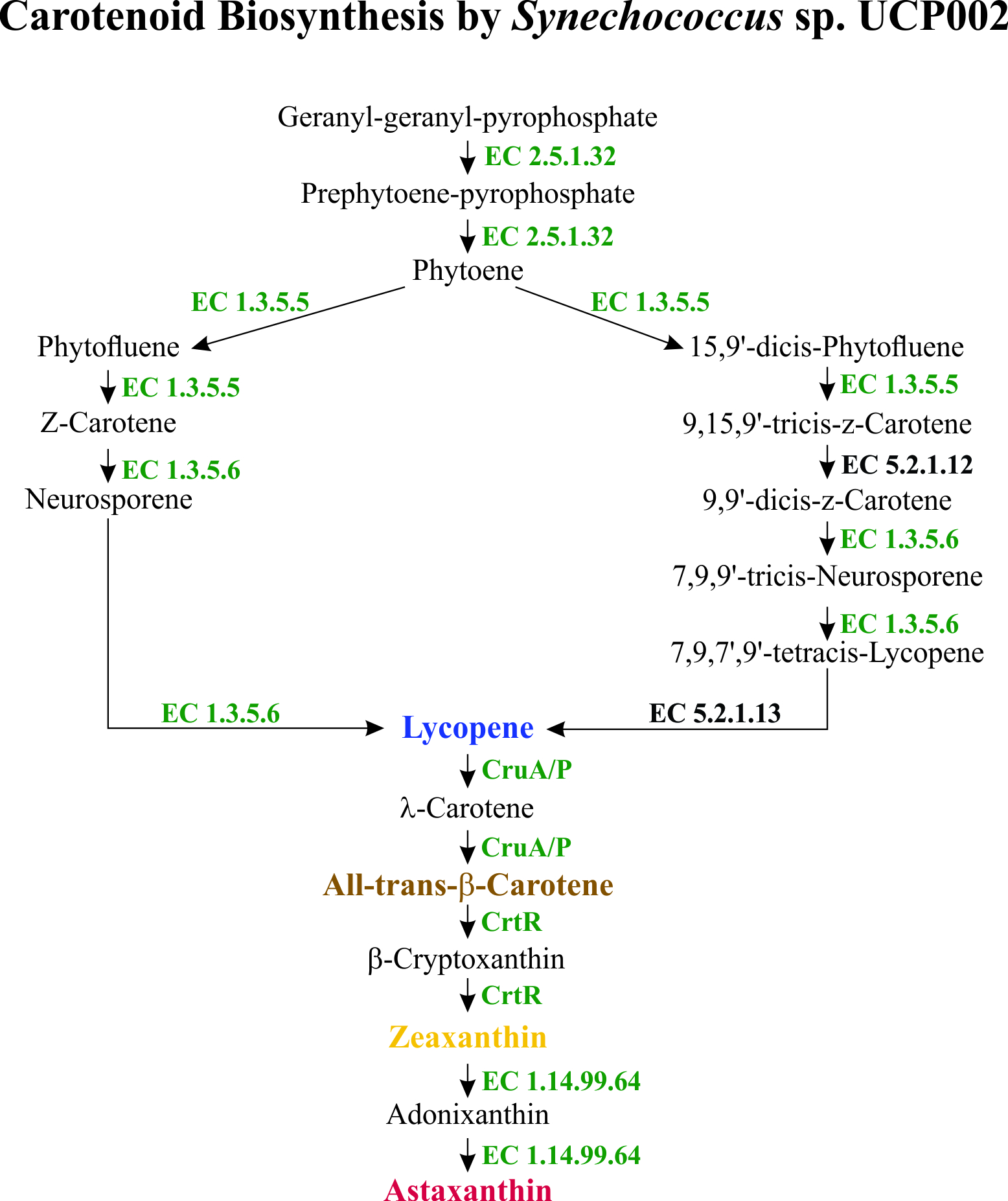

Supplement: Supplementary file 6 [file Image4.JPEG]

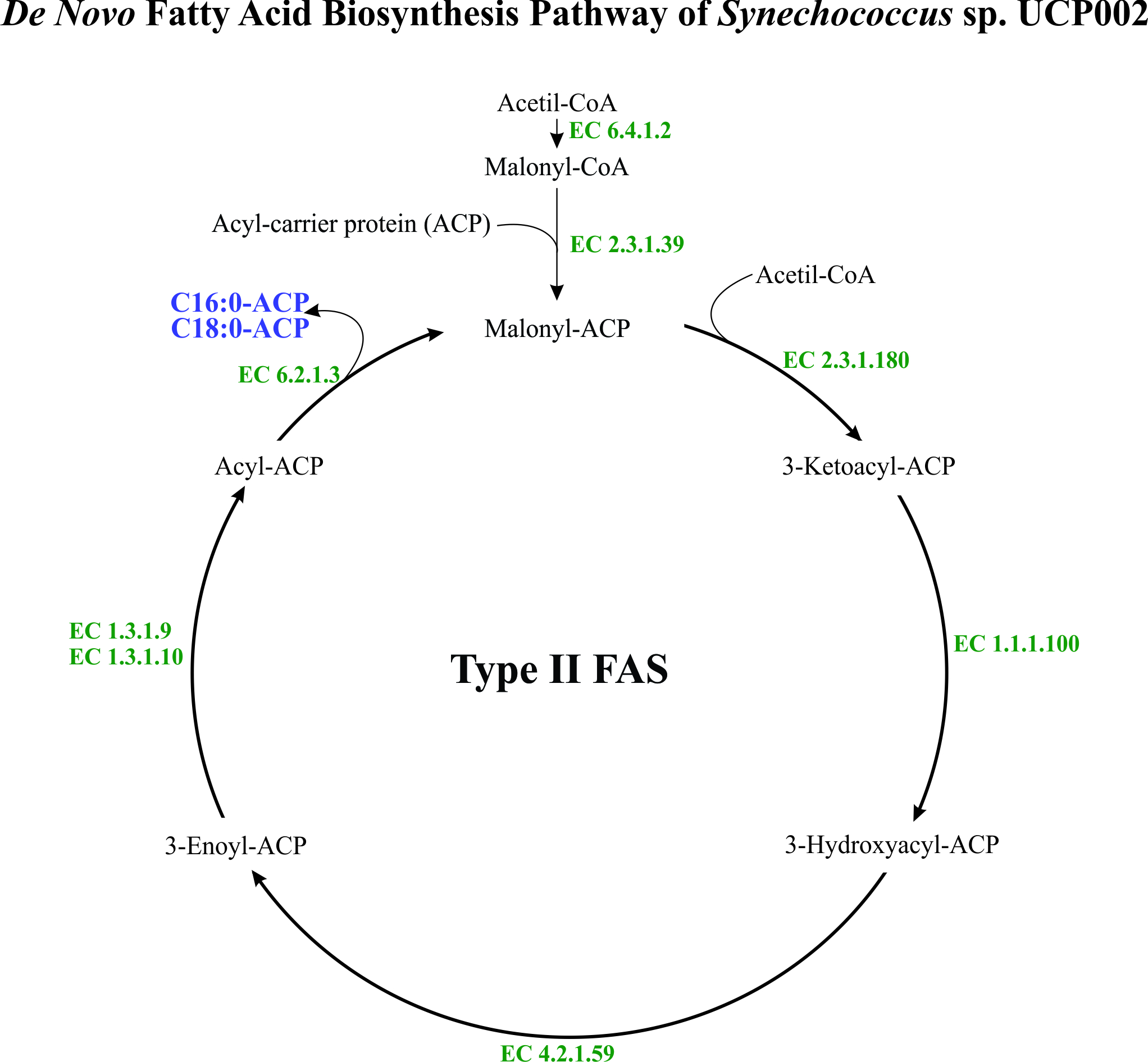

Supplement: Supplementary file 7 [file Image7.JPEG]

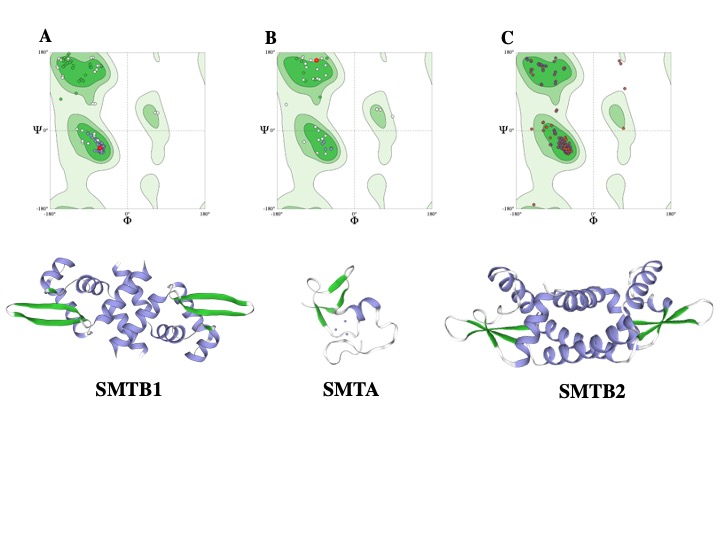

Supplement: Supplementary file 8 [file Image2.JPEG]

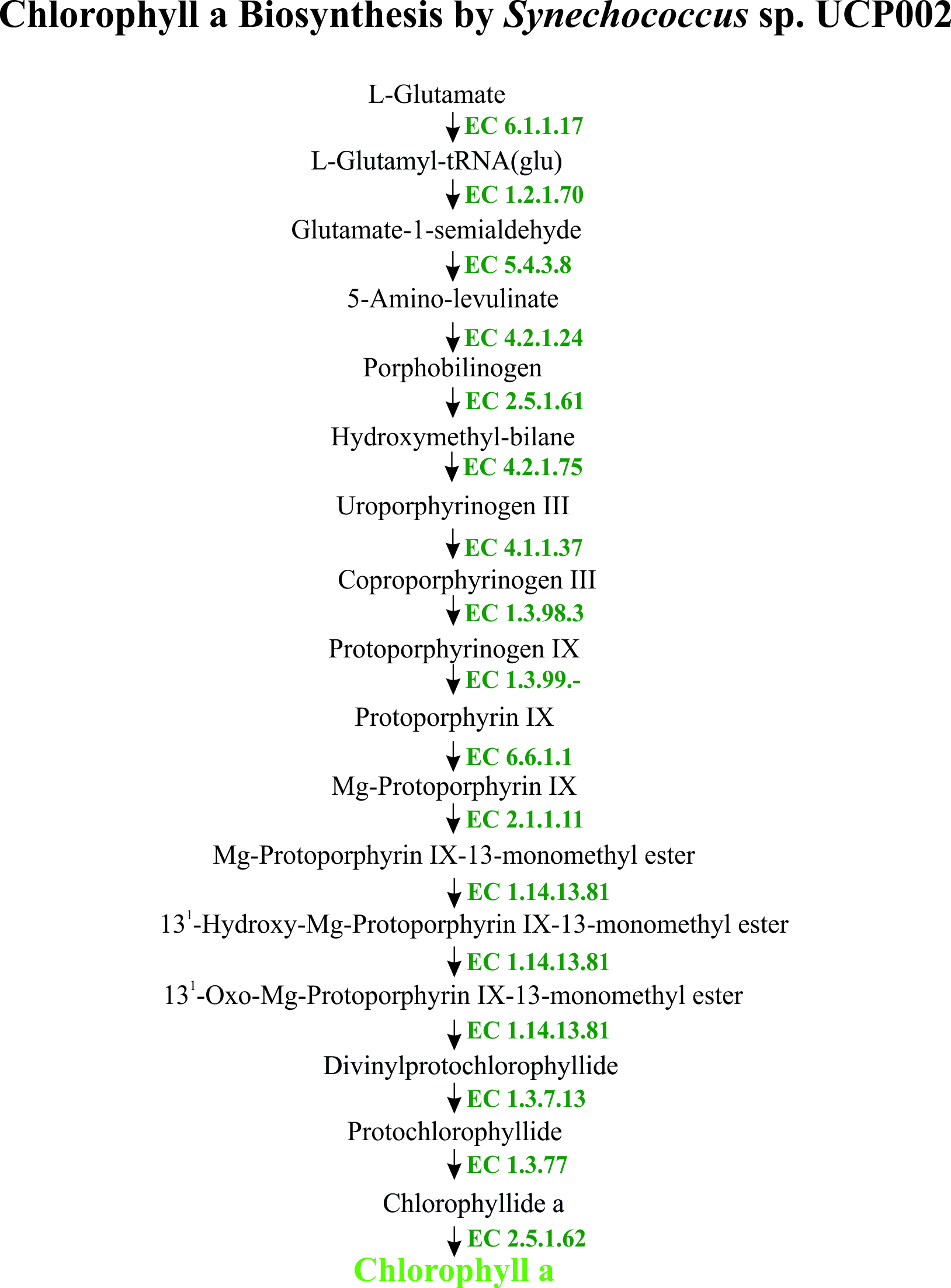

Supplement: Supplementary file 9 [file Image5.JPEG]

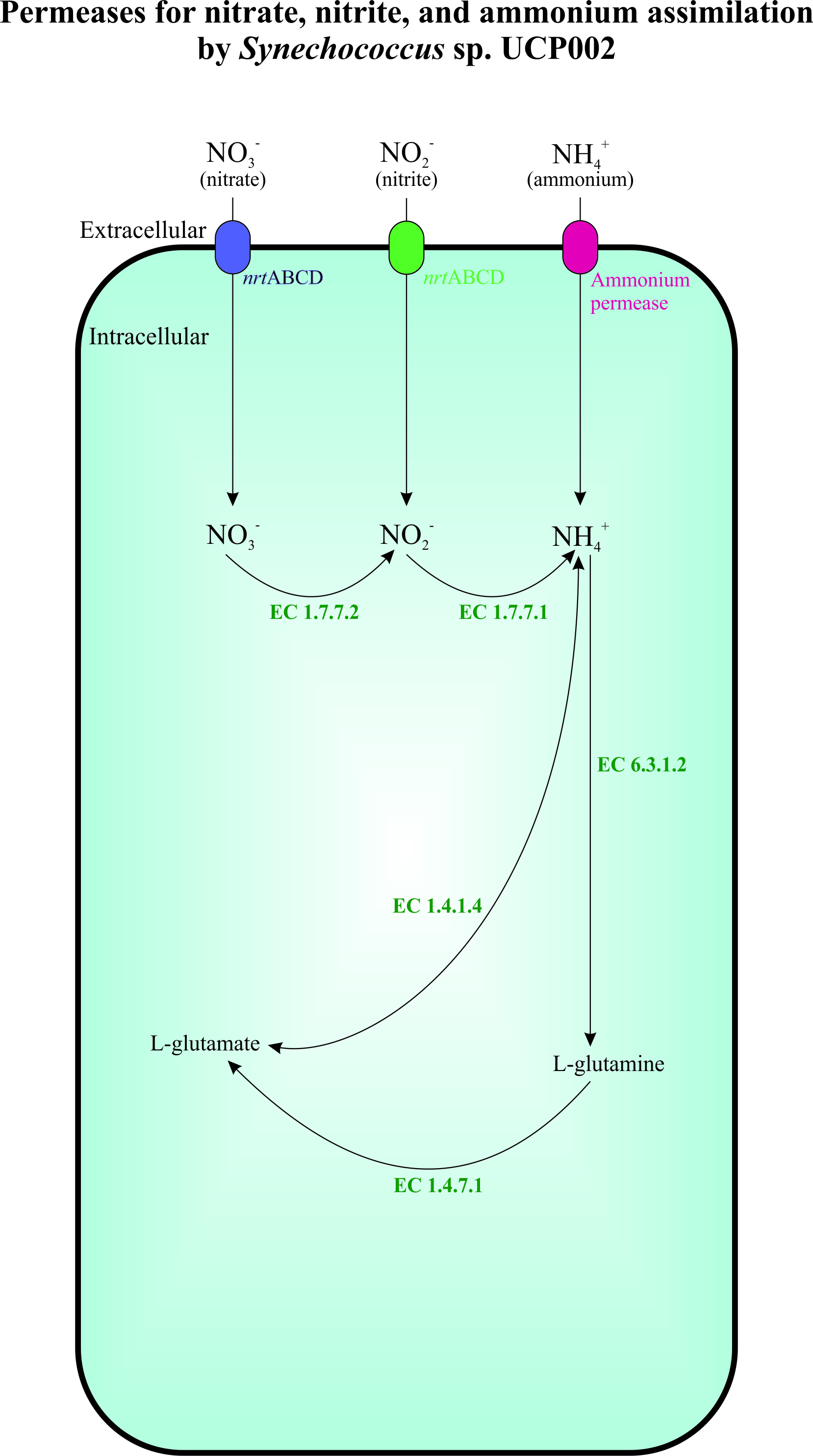

Supplement: Supplementary file 11 [file Image8.JPEG]

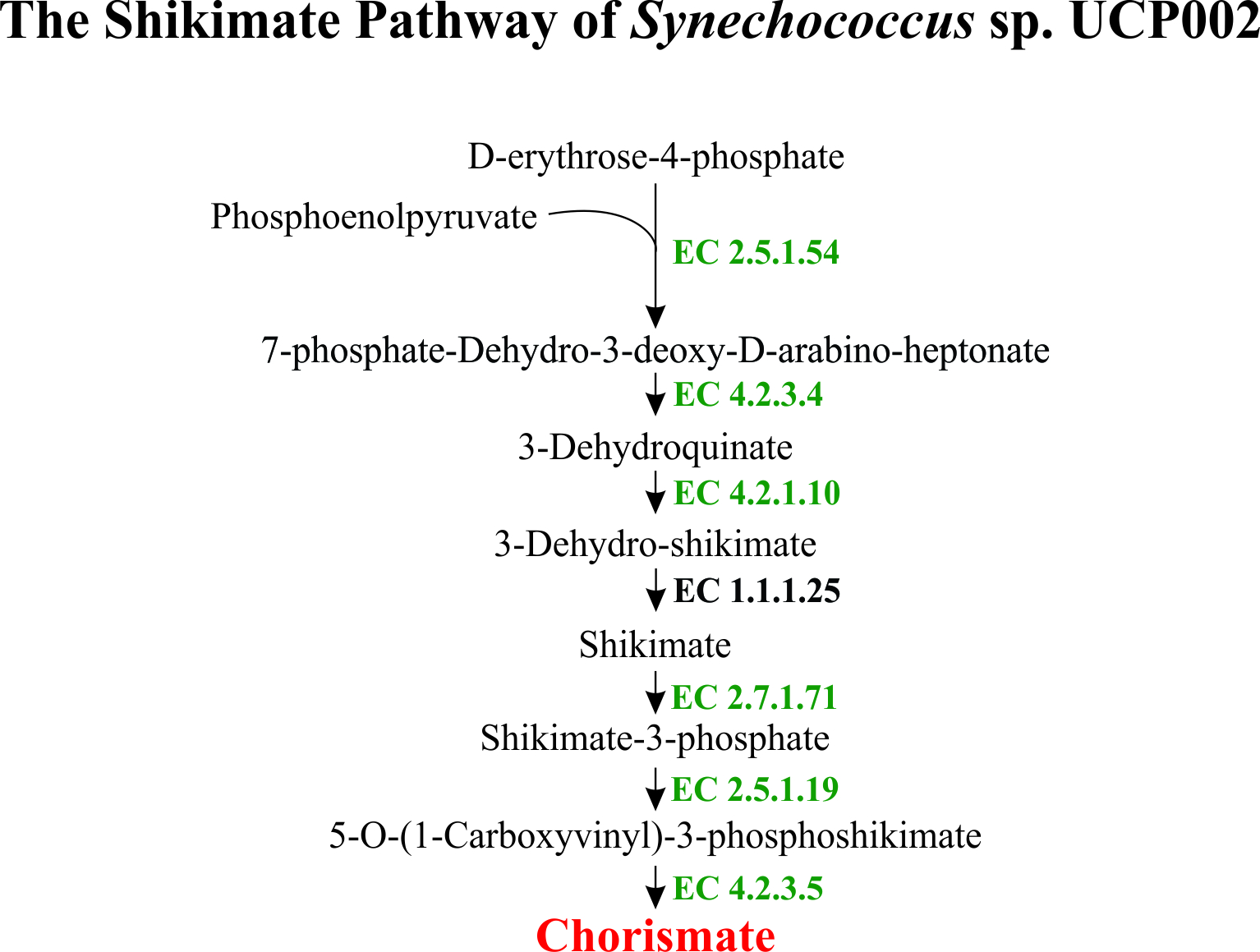

Supplement: Supplementary file 12 [file Image6.JPEG]
